# Tumor-derived neomorphic mutations in ASXL1 impairs the BAP1-ASXL1-FOXK1/K2 transcription network

**DOI:** 10.1007/s13238-020-00754-2

**Published:** 2020-07-18

**Authors:** Yu-Kun Xia, Yi-Rong Zeng, Meng-Li Zhang, Peng Liu, Fang Liu, Hao Zhang, Chen-Xi He, Yi-Ping Sun, Jin-Ye Zhang, Cheng Zhang, Lei Song, Chen Ding, Yu-Jie Tang, Zhen Yang, Chen Yang, Pu Wang, Kun-Liang Guan, Yue Xiong, Dan Ye

**Affiliations:** 1 Huashan Hospital, Fudan University, and Molecular and Cell Biology Lab, Institutes of Biomedical Sciences, and the Shanghai Key Laboratory of Medical Epigenetics, and the Key Laboratory of Metabolism and Molecular, Ministry of Education, Shanghai, 200032 China; 2 The International Co-Laboratory of Medical Epigenetics and Metabolism, Ministry of Science and Technology, Shanghai, 200032 China; 3 Department of Neurosurgery, Huashan Hospital, Fudan University, Shanghai, 200032 China; 4 Key Laboratory of Cell Differentiation and Apoptosis of National Ministry of Education, Department of Pathophysiology, Shanghai Jiao Tong University School of Medicine, Shanghai, 200025 China; 5 Key Laboratory of Synthetic Biology, Institute of Plant Physiology and Ecology, Shanghai, 200032 China; 6 Institutes for Biological Sciences, Chinese Academy of Sciences, Shanghai, 200032 China; 7 State Key Laboratory of Proteomics, Beijing Proteome Research Center, Beijing Institute of Radiation Medicine, Beijing, 102206 China; 8 National Center for Protein Sciences (The PHOENIX Center, Beijing), Beijing, 102206 China; 9 State Key Laboratory of Genetic Engineering and Collaborative Innovation Center for Genetics and Development, School of Life Sciences, Institutes of Biomedical Sciences, Fudan University, Shanghai, 200032 China; 10 Department of Pharmacology and Moores Cancer Center, University of California San Diego, La Jolla, CA USA; 11 Lineberger Comprehensive Cancer Center, Department of Biochemistry and Biophysics, University of North Carolina, Chapel Hill, NC USA; 12 Department of General Surgery, Huashan Hospital, Fudan University, Shanghai, 200032 China

**Keywords:** ASXL1, BAP1, FOXK1/K2, leukemia, epigenetics

## Abstract

Additional sex combs-like 1 (ASXL1) interacts with BRCA1-associated protein 1 (BAP1) deubiquitinase to oppose the polycomb repressive complex 1 (PRC1)-mediated histone H2A ubiquitylation. Germline *BAP1* mutations are found in a spectrum of human malignancies, while *ASXL1* mutations recurrently occur in myeloid neoplasm and are associated with poor prognosis. Nearly all *ASXL1* mutations are heterozygous frameshift or nonsense mutations in the middle or to a less extent the C-terminal region, resulting in the production of C-terminally truncated mutant ASXL1 proteins. How ASXL1 regulates specific target genes and how the C-terminal truncation of *ASXL1* promotes leukemogenesis are unclear. Here, we report that ASXL1 interacts with forkhead transcription factors FOXK1 and FOXK2 to regulate a subset of FOXK1/K2 target genes. We show that the C-terminally truncated mutant ASXL1 proteins are expressed at much higher levels than the wild-type protein in *ASXL1* heterozygous leukemia cells, and lose the ability to interact with FOXK1/K2. Specific deletion of the mutant allele eliminates the expression of C-terminally truncated ASXL1 and increases the association of wild-type ASXL1 with BAP1, thereby restoring the expression of BAP1-ASXL1-FOXK1/K2 target genes, particularly those involved in glucose metabolism, oxygen sensing, and JAK-STAT3 signaling pathways. In addition to FOXK1/K2, we also identify other DNA-binding transcription regulators including transcription factors (TFs) which interact with wild-type ASXL1, but not C-terminally truncated mutant. Our results suggest that ASXL1 mutations result in neomorphic alleles that contribute to leukemogenesis at least in part through dominantly inhibiting the wild-type ASXL1 from interacting with BAP1 and thereby impairing the function of ASXL1-BAP1-TF in regulating target genes and leukemia cell growth.

**Electronic supplementary material:**

The online version of this article (10.1007/s13238-020-00754-2) contains supplementary material, which is available to authorized users.

## INTRODUCTION

Addition of sex combs (*Asx*) gene was originally discovered from genetic screen in *Drosophila* where *Asx* mutations augmented the phenotype of both trithorax (*TrxG*) and polycomb group (*PcG*) gene mutants (Sinclair et al., 1998). Subsequent discoveries that *TrxG* and *PcG* genes encode histone modifying enzymes link the function of Asx to epigenetic and transcriptional regulation. Human genome contains three *Asx*-like genes, *ASXL1-3*. Mammalian ASXL proteins share a common domain architecture, consisting of a C-terminally located PHD domain that recognizes methylated histone tail and is conserved in *Drosophila* Asx protein (Micol and Abdel-Wahab, 2016). Mammalian ASXL proteins uniquely contain an N-terminal HARE-HTH domain (residues 1–94 in human ASXL1, also known as ASXN domain) that was found in HB1, ASXL, restriction endonuclease and formed as winged helix-turn-helix (HTH) fold, and a DEUBAD (DEUBiquitinase adaptor) domain (residues 238–390, also known as ASXH domain) shared with ubiquitin carboxyl-terminal hydrolase 37 (Uch37) and transcription factor NF-κB (Sanchez-Pulido et al., 2012).

ASXL proteins do not contain a catalytic domain and instead, they participate in epigenetic regulation through interacting with other histone modifying enzymes. The major functional and physical partner of ASXL proteins is tumor suppressor BRCA1-associated protein 1 (BAP1), also known as Calypso in *Drosophila* (Scheuermann et al., 2010; Dey et al., 2012), a UCH domain-containing deubiquitylase. BAP1 catalyzes the removal of monoubiquitin from histone H2A lysine 119 (H2AK119 monoubiquitination). ASXL1 binds to BAP1 and stimulates BAP1’s substrate binding affinity and activity to oppose PRC1 (polycomb repressive complex 1)-catalyzed H2AK119 monoubiquitylation, thereby releasing the polycomb repression (Scheuermann et al., 2010; Dey et al., 2012; Sahtoe et al., 2016). A recent study found that there was a large overlap in genes downregulated between BAP1 knockout (KO) and ASXL1/2 double KO cells (Campagne et al., 2019), supporting the important role of ASXL proteins in mediating the function of BAP1.

**Figure 1:**
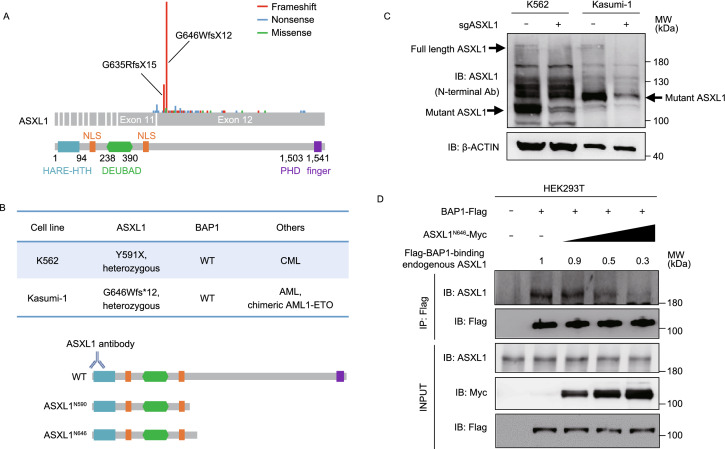

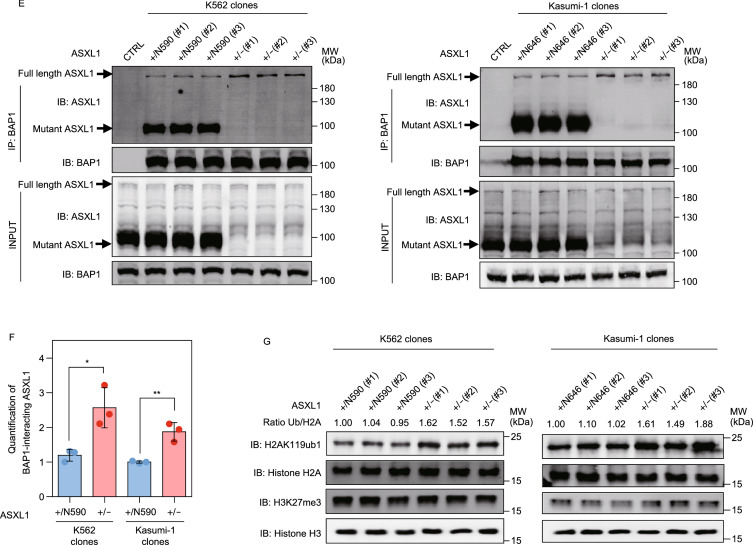
**Tumor-derived C-terminally truncated ASXL1 mutants compete with wild-type ASXL1 for binding with BAP1**. (A) AML-derived mutations in *ASXL1* gene include frameshift mutations (in red), nonsense (in blue), and missense mutations (in green), modified from a previous study by Schnittger et al. NLS, nuclear localization signal; HARE-HTH, HB1, ASXL, restriction endonuclease Helix-Turn-Helix domain; DEUBAD, DEUBiquitinase adaptor domain; PHD, plant homedomain finger. (B) *ASXL1* mutation status in K562 and Kasumi-1 cells, and schematic representation of wild-type ASXL1, Y591X and G646Wfs*12 mutations, and the antibody specific to the N-terminal sequence of ASXL1 protein. See also Fig. S1A and S1B. (C) Western blot analysis of the endogenous proteins of full-length and C-terminally truncated mutant ASXL1 in K562 and Kasumi-1 cells. (D) Increased amount of ASXL1^N646^-Myc were overexpressed in HEK293T cells together with BAP1-Flag, and the interaction between BAP1-Flag and endogenous ASXL1 was determined by Western blot analysis. Relative Flag-BAP1-binding ASXL1 protein levels were quantified. (E) Cells with deletion of mutant *ASXL1* allele (referred to as *ASXL1*^+/−^) were generated using the CRISPR/Cas9 system in leukemia cells of K562 (left) and Kausmi-1 (right). Co-IP was performed to detect the interaction between BAP1 and ASXL1. (F) Quantification of the protein levels of BAP1-interacting ASXL1 in (E) is shown. (G) Global H2AK119 mono-ubiquitination and H3K27 trimethylation levels in *ASXL1*^+/N590^ and *ASXL1*^+/−^ cells in K562 (left) and *ASXL1*^+/N646^ and *ASXL1*^+/−^ cells in Kausmi-1 (right). Relative H2AK119 mono-ubiquitination was normalized by Histone H2A. Asterisks denote statistical significance with two-tailed Student’s *t*-test. *P < 0.05 and **P < 0.01 for the indicated comparison

Complete loss of *Asxl1* in mice impairs hematopoiesis and causes developmental abnormalities including dwarfism, anophthalmia, and near-penetrant embryonic lethality, whereas the surviving *Asxl1*^−/−^ mice or hematopoietic-specific deletion of *Asxl1* had shortened lifespan and developed MDS features, but not full-blown myelodysplasia or leukemia (Fisher et al., 2010). Nearly all mutations targeting three *ASXL* genes found in human myeloid malignancies are heterozygous frameshift or nonsense mutations targeting either exon 11 or 12 (Schnittger et al., 2013; Micol and Abdel-Wahab, 2016; as summerized in Fig. 1A), resulting in prematurely truncated variants of ASXL that retain the N-terminal 400 residues or so that include both HARE-HTH and DEUBAD domains (Schnittger et al., 2013; Micol and Abdel-Wahab, 2016). The C-terminally truncated ASXL1 proteins were detected in tumor cells and were sufficient to promote the deubiquitylase activity of BAP1 (Balasubramani et al., 2015; Inoue et al., 2016; Asada et al., 2018). Forced expression of C-terminally truncated Asxl1 mutant in mice generated myeloid malignancies (Asada et al., 2018) and MDS-like diseases (Inoue et al., 2013), and promoted susceptibility to leukemic transformation (Nagase et al., 2018). Conditional knock-in of a tumor-derived mutation generating a C-terminally truncated Asxl1 mutant led to myeloid skewing, age-dependent anemia, thrombocytosis, and morphological dysplasia in mice (Nagase et al., 2018). These studies support a potential oncogenic role of C-terminally truncated ASXL1 in perturbing hematopoiesis and promoting susceptibility to leukemic transformation. How ASXL1 mutations contribute to myeloid malignancies is not fully understood and this study aims to address this issue.

## RESULTS

### Tumor-derived C-terminally truncated ASXL1 mutants compete with wild-type ASXL1 for binding with BAP1

The leukemia cell lines K562 and Kasumi-1 harbor endogenous ASXL1 mutations, generating a nonsense mutation Y591X and a frameshift mutation G646Wfs*12, leading to the premature termination of ASXL1 protein at codon 590 and 646, respectively (Figs. 1B, S1A and S1B). We therefore refer to these cells as *ASXL1*^+/N590^ K562 and *ASXL1*^+/N646^ Kasumi-1, respectively. Notably, the protein expression of C-terminally truncated mutant ASXL1 was much higher than that of full-length ASXL1 in both types of leukemia cells (Fig. 1C). Flag-tagged N-terminal ASXL1 (residues 1 to 646, referred to as ASXL1^N646^) exhibited increased protein stability than wild-type ASXL1 when overexpressed in HEK293T cells upon treatment with cycloheximide (CHX), a common reagent used to inhibit protein synthesis (Fig. S2A). CHX-chase experiments in *ASXL1*^+/N590^ K562 also showed that endogenous protein of mutant ASXL1 was more stable than wild-type protein, while their mRNA expression levels were comparable (Fig. S2B and S2C). These results are consistent with a previous finding that BAP1 binding stabilizes mutant ASXL1 more effectively than the wild-type ASXL1 (Asada et al., 2018).

Both full-length and C-terminally truncated mutant ASXL1 have been reported to interact with BAP1 (Balasubramani et al., 2015; Asada et al., 2018), which prompted us to test whether mutant ASXL1 would compete with wild-type ASXL1 to bind with BAP1 as mutant ASXL1 protein was at a much higher level. Our data demonstrated that ASXL1^N646^-Myc dose-dependently reduced the protein association of Flag-tagged BAP with endogenous ASXL1 in HEK293T cells (Fig. 1D). Importantly, we found that genetic deletion of the C-terminally truncated *ASXL1* allele (referred to as *ASXL1*^+/−^), significantly increased the endogenous protein association of wild-type ASXL1 with BAP1 by as much as 2 folds compared to the corresponding control *ASXL1*^+/N590^ K562 and *ASXL1*^+/N646^ Kasumi-1 (Fig. 1E and 1F).

It has been reported that mutant ASXL1 proteins gain new functions, including enhancing the catalytic activity of BAP1 and thereby reducing H2AK119 mono-ubiquitination (Balasubramani et al., 2015; Sahtoe et al., 2016; Asada et al., 2018). In accord, we found that global H2AK119 mono-ubiquitination levels were increased in *ASXL1*^+/−^ cells compared to *ASXL1*^+/N590^ K562 and *ASXL1*^+/N646^ Kasumi-1 (Fig. 1G). Moreover, we found that global H3K27me3 levels were comparable between *ASXL1*^+/−^ and *ASXL1*^+/N590^ or *ASXL1*^+/N646^ cells (Fig. 1G), in line with a previous study showing that unlike the effect of complete *Asxl1* loss, knock-in a C-terminally truncated Asxl1 mutant led to a substantial reduction in H2AK119 mono-ubiquitination without affecting H3K27me3 (Abdel-Wahab et al., 2013).

Taken together, these findings suggest that C-terminally truncated mutant ASXL1 proteins are highly expressed in leukemia cells and impair the association of wild-type ASXL1 with BAP1 in a dominant-negative manner.

### C-terminally truncated ASXL1 mutants retain BAP1 binding, but lose interaction with FOXK1 and FOXK2

**Figure 2:**
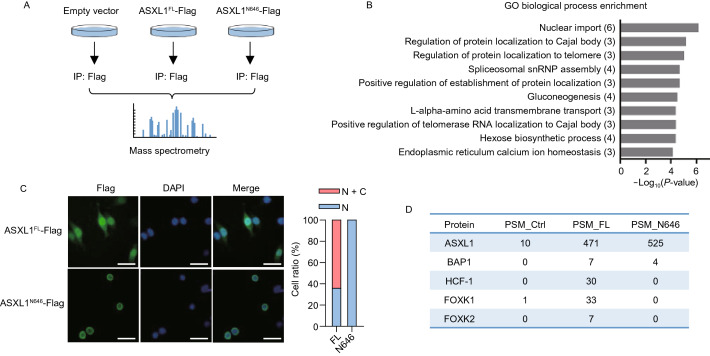

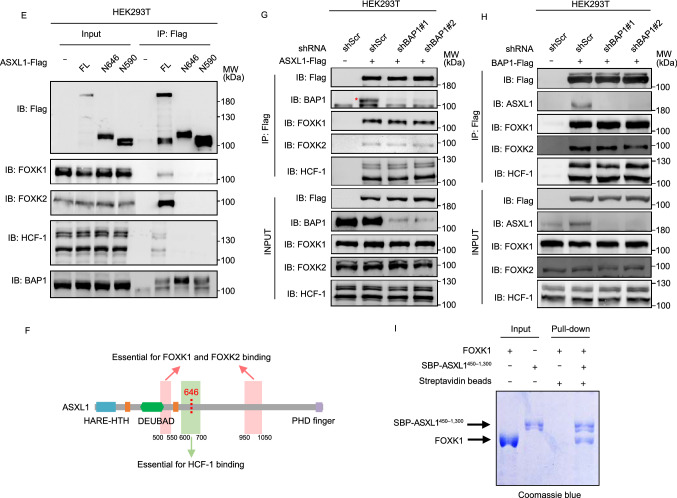
**C-terminally truncated ASXL1 mutants retain BAP1 binding, but impair or lose interaction with HCF-1 and FOXK1/2**. (A) Cartoon representation of the immunoprecipitation–Mass Spectrometry (IP-MS) assay for identification of interacting proteins of full-length ASXL1 (ASXL1^FL^-Flag) and tumor-derived mutant ASXL1 (ASXL1^N646^-Flag). (B) Gene Ontology (GO) biological process enrichment analysis of proteins that lose the interaction with C-terminally truncated ASXL1 mutant as identified by IP-MS. (C) Localization of ASXL1^FL^-Flag and ASXL1^N646^-Flag in HEK293T cells. (D) IP-MS identification of interaction between ASXL1^N646^-Flag and endogenous proteins of BAP1, HCF-1, FOXK1, and FOXK2 in U2OS cells, as described above in (A). PSM, the total number of identified peptide spectra matched for the protein. (E) Interaction between different ASXL1 truncations and endogenous HCF-1, FOXK1, FOXK2 and BAP1. See also Fig. S2A. (F) Domain mapping of ASXL1 reveals the residues in ASXL1 which are essential for FOXK1, FOXK2, HCF-1 binding. See also Fig. S3. (G) Interaction between full-length ASXL1 and endogenous HCF-1, FOXK1 and FOXK2 in *BAP1* knockdown cells. (H) Interaction between BAP1 and endogenous HCF-1, FOXK1 and FOXK2 in *ASXL1* knockdown cells. (I) *In vitro* pull-down assay shows ASXL1 interacts with FOXK1 directly

To elucidate the mechanisms underlying the function of wild-type ASXL1 and the oncogenic role of C-terminally truncated mutant ASXL1 proteins, we set out to identify the interacting proteins of wild-type ASXL1 or tumor-derived ASXL1^N646^ (Fig. 2A). To this end, a number of proteins were found to bind with wild-type ASXL1, but not ASXL1^N646^ (Table S1). Gene Ontology (GO) analysis indicated that ASXL1^N646^ lost interaction with proteins associated with nuclear transport (Fig. 2B), raising the possibility that C-terminal-truncating mutations might change the subcellular localization of ASXL1. Indeed, immunofluorescence staining demonstrated that ASXL1-Flag protein localized to both the nucleus and the cytoplasm, whereas C-terminally truncated ASXL1^N646^-Flag localized predominantly to the nucleus (Fig. 2C).

It has previously been reported that BAP1 forms a polycomb repressive deubiquitylase (PR-DUB) with the HCFC1 transcriptional scaffolding subunit (host cell factor-1, also called HCF-1) and DNA-sequence specific transcription factors FOXK1 and FOXK2 (White and Harper, 2012). In agreement, we found ASXL1-Flag interacted with endogenous BAP1, HCF-1, and FOXK1/K2 in U2OS (Fig. 2D). Despite its predominant nuclear localization (Fig. 2C) and ability to interact with BAP1, ASXL1^N646^-Flag lost interaction with endogenous HCF-1 and FOXK1/K2 (Fig. 2D). When overexpressed in HEK293T cells, Flag-tagged wild-type ASXL1, but not C-terminally truncated N590, N646, G646Wfs*12 mutants or N-terminal-truncated protein (∆646), could interact with endogenous HCF-1 and FOXK1/K2 (Figs. 2E and S3). When ASXL1^N646^-Flag was overexpressed at very high levels, its weak interaction with endogenous HCF-1 could be detected in HEK293T cells (Fig. S4A), suggesting that C-terminally truncation mutants of ASXL1 may substantially impair, if not completely abolish, the association with HCF-1.

Next, domain mapping of ASXL1 revealed that residues 600 to 700 in ASXL1 were crucial for HCF-1 binding, while two regions in ASXL1 (residues 500 to 550 and residues 950 to 1,050) were essential for FOXK1 and FOXK2 binding (Figs. 2F, S4B and S5A–D). Most likely, the interaction between FOXK1/K2 and ASXL1 requires both regions, and either of these two domains in ASXL1 solely is not sufficient to mediate the protein association with FOXK1/K2. Moreover, we observed that Flag-ASXL1 interacted with endogenous HCF-1, FOXK1 and FOXK2 in *BAP1* knockdown HEK293T cells (Fig. 2G), and that Flag-BAP1 interacted with endogenous HCF-1, FOXK1, and FOXK2 in *ASXL1* knockdown HEK293T cells (Fig. 2H). Furthermore, *in vitro* studies with purified recombinant proteins demonstrated a direct interaction between ASXL1 and FOXK1 proteins (Fig. 2I).

Together, these data suggest that wild-type ASXL1 forms a complex with HCF-1 and FOXK1/K2 independent of BAP1, and that the C-terminally truncated mutant ASXL1 can bind to BAP1 but loses interaction with FOXK1/K2.

### C-terminally truncated ASXL1 mutants lose interaction with multiple DNA-binding transcription regulators

**Figure 3:**
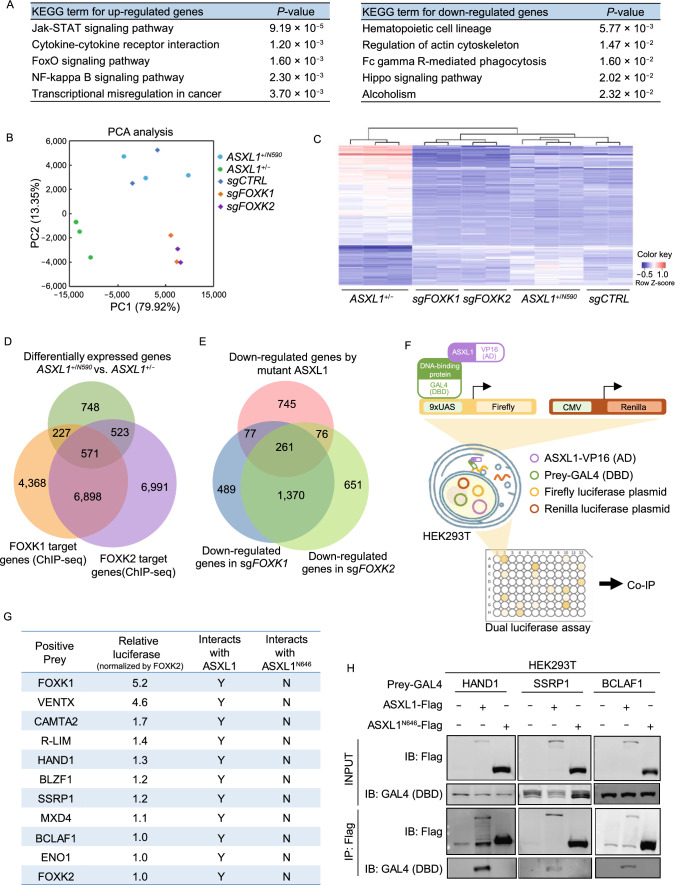
**C-terminally truncated ASXL1 mutants lose interaction with multiple DNA-binding proteins**. (A) RNA-seq analysis reveals differentially expressed genes by deletion of mutant ASXL1 in K562 cells (*ASXL1*^+/N590^ vs. *ASXL1*^+/−^). (B) Principal component analysis (PCA) of RNA-sequencing results among the indicated groups of K562 cells. (C) Heatmap of 231 genes differentially expressed in the indicated groups of K562 cells, as identified by RNA-seq and analyzed by two-way ANOVA analysis. (D) Venn diagrams depicting a significant proportion of genes differentially affected by deletion of mutant ASXL1 are direct target genes of FOXK1 or FOXK2 (fold change > 1.4). (E) Venn diagrams depicting overlap between the indicated groups of K562 cells, including the overlap between down-regulated genes by mutant ASXL1 (*n* = 1,159) and down-regulated genes by deletion of either FOXK1 (*n* = 2,197) or FOXK2 (*n* = 2,358), as determined by RNA-seq analysis. (F) Schematic representation of the mammalian two-hybrid system. (G and H) A summary of DNA-binding proteins which selectively bind with full-length ASXL1, but not ASXL1^N646^, identified by the mammalian two-hybrid system and verified by Western blot. Some representative Western blot results are shown in (H). See also Fig. S7B–E

To determine the functional consequence of heterozygous expression of C-terminally truncated mutant ASXL1, we profiled gene expression in *ASXL1*^+/N590^ and *ASXL1*^+/−^ K562 cells, and found that 2,069 genes were differentially expressed (*P* < 0.05; fold-change > 1.5), including 1,184 down-regulated and 1,159 up-regulated genes in *ASXL1*^+/−^ compared to *ASXL1*^+/N590^ cells (Table S2). Kyoto Encyclopedia of Genes and Genomes (KEGG) analysis showed that deletion of mutant *ASXL1* allele significantly (*P* < 0.01) up-regulated genes enriched in JAK-STAT signaling pathway, Cytokine-cytokine receptor interaction, FoxO signaling pathway, NF-kappa B signaling pathway, and transcriptional misregulation in cancer, while significantly (*P* < 0.05) down-regulated genes were enriched in hematopoietic cell lineage, regulation of actin cytoskeleton, Fc gamma R-mediated phagocytosis, Hippo signaling pathway and alcoholism (Fig. 3A and Table S3).

FOXK1 and FOXK2 function as sequence-specific DNA binding factors for the BAP1-containing PR-DUB complex (White and Harper, 2012; Ji et al., 2014), and are involved in the regulation of multiple cellular pathways, such as cell signaling (Gao, 2005), proliferation (Williamson et al., 2005), and metabolism (Sukonina et al., 2019). In stable K562 cell pools with FOXK1/K2 depletion (Fig. S6A and S6B), our RNA-seq analysis revealed that of differentially expressed genes, 62.5% (2,197 of 3,518) and 66% (829 of 3,573) were significantly (*P* < 0.05; fold-change > 1.2) down-regulated by depletion of FOXK1 and FOXK2, respectively (Fig. S6C and S6D). A large proportion of down-regulated genes in sg*FOXK1* cells overlapped with those in sg*FOXK2* cells (Fig. S6E), and the overlapped gene were enriched in hematopoietic stem cell differentiation, integrin activation, regulation of hormone biosynthetic process and monoamine transport (Fig. S6F), suggesting that FOXK1 and FOXK2 are functionally overlapping in regulating their target genes.

Principal component analysis (PCA) and unsupervised hierarchical cluster analysis of RNA-Seq data illustrated that *ASXL1*^+/N590^ and *ASXL1*^+/−^ clones were clearly separated, and that sg*FOXK1* and sg*FOXK2* samples were clustered together (Fig. 3B and 3C), supporting the effect of C-terminally truncated mutant ASXL1 on regulating genes as well as the functional similarity of FOXK1 and FOXK2 in regulating their targets. By comparing the FOXK1 and FOXK2 chromatin immunoprecipitation assays with sequencing (ChIP-seq) data, we found that 1,321 of 2,069 (63.8%) genes, which were affected by deletion of mutant *ASXL1* allele, were bound by FOXK1 and/or FOXK2 (Fig. 3D). Among 1,159 genes down-regulated by mutant *ASXL1*, 414 of them (35.7%) were down-regulated in sg*FOXK1* or sg*FOXK2* cells (Fig. 3E), supporting that ASXL1 mutants interfere with the ASXL1-BAP1-FOXK1/K2 axis and regulate a significant fraction of downstream targets of FOXK1 and FOXK2.

Will the ASXL1-BAP1 complex regulate downstream gene expression via interaction with other TFs, in addition to FOXK1 and FOXK2? To address this question, we carried out a mammalian two-hybrid screen of a human TF library containing 1,401 known or putative DNA binding proteins, and examined their interaction with ASXL1 (Fig. 3F). As expected, both FOXK1 and FOXK2 were identified to be positive prey proteins interacting with ASXL1-VP16, with the relative luciferase activity of FOXK2-ASXL1 interaction being lower than that of FOXK1-ASXL1 interaction (Fig. 3G). We therefore arbitrarily set the relative luciferase activity of FOXK2-ASXL1 interaction as “1” and selected those factors displaying equal or higher luciferase activity for further characterization. By using this criterion, we identified additional 26 potential ASXL1-interacting proteins (Table S4), and then verified their protein association with ASXL1 by Co-IP and Western blot (Figs. 3H and S7A–E). Moreover, we found at least 9 positive prey proteins which solely interacted with ASXL1, but not ASXL1^N646^ (Fig. 3G and 3H), indicating that the C-terminally truncated mutant ASXL1 may interfere with the axis of BAP1-ASXL1-DNA-binding transcription regulators to control gene expression.

### C-terminally truncated ASXL1 mutant interferes with the BAP1-ASXL1-FOXK1/K2 axis to down-regulate multiple tumor suppressor genes

**Figure 4:**
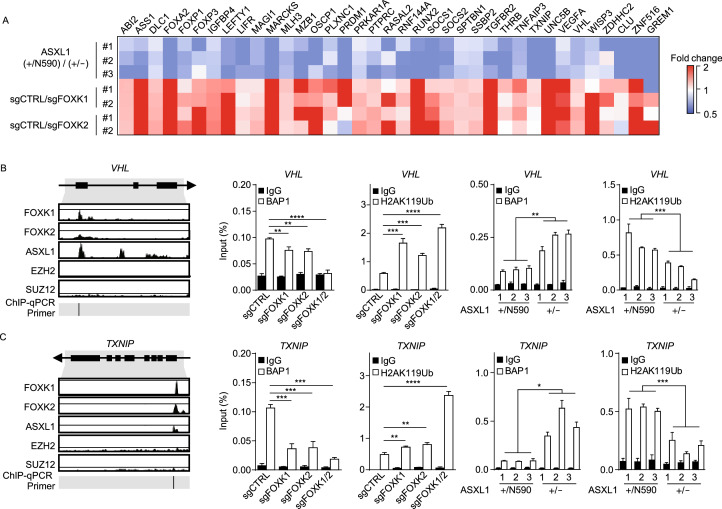

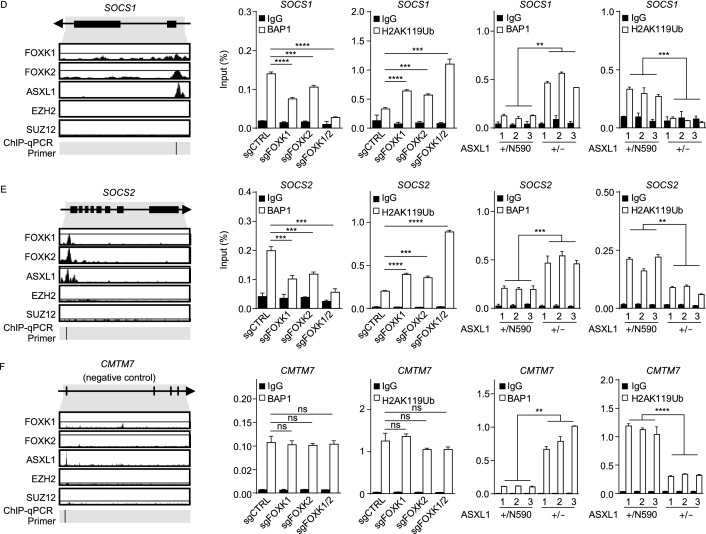
**Tumor-derived ASXL1 mutant interferes with the BAP1-ASXL1-FOXK1/K2 axis to down-regulate tumor suppressor genes**. (A) The mRNA expression of tumor suppressor genes in the indicated groups of K562 cells, as determined by RNA-seq analysis. (B–F) The occupancy of FOXK1, FOXK2, ASXL1, EZH2 and SUZ12 at the promoter regions of indicated genes, analyzed by using the Cistrome Data Browser and UCSC genome browser. Moreover, BAP1 and H2AK119Ub enrichment at promoters of these genes were detected by ChIP-qPCR in *ASXL1*^+/N590^ knockout K562 cells and *FOXK1* and/or *FOXK2* knockout K562 pools. IgG was included as negative control for ChIP-qPCR. According to ChIP-Seq retrieved from the GTRD database (https://gtrd.biouml.org/), *CMTM7* is not a direct target gene of FOXK1 and FOXK2, although BAP1 is enriched at the promoter region of *CMTM7.* This gene thus is included as a negative control. Asterisks denote statistical significance with two-tailed Student’s *t*-test or one-way ANOVA (B–F). *P < 0.05; **P < 0.01; ***P < 0.001; ****P < 0.0001 for the indicated comparison; ns = not significant

Among the genes whose expression was down-regulated by C-terminally truncated ASXL1 mutation or knocking-down of *FOXK1* or *FOXK2* are multiple tumor suppressors, including Von Hippel-Lindau syndrome (*VHL*), thioredoxin interacting protein (*TXNIP*), suppressor of cytokine signaling 1 and 2 (*SOCS1*, *SOCS2*), membrane associated guanylate kinase 1 (*MAGI1*) and zinc finger protein 516 (*ZNF516*) (Fig. 4A). Analyses of the TCGA data retrieved from cBioPortal revealed a positive correlation between the expression of *BAP1 a*nd that of FOXK1/K2 direct target genes in a panel of human cancer cell lines, including *VHL* and *SOCS1*/*2* (Fig. S8), supporting these tumor suppressor genes are downstream targets of the BAP1-containing PR-DUB complex.

According to previous ChIP-seq data (Fig. S9A), FOXK1, FOXK2, and ASXL1 co-bound the same promoter regions of their target genes (e.g., *VHL*, *TXNIP*, *SOCS1*, *SOCS2*, *MAGI1* and *ZNF516*), whereas enhancer of zeste homolog 2 (EZH2) and suppressor of zeste 12 (SUZ12), two core components of the PRC2 complex, did not bind to these regions (Figs. 4B–E, S9B and S9C). Neither FOXK1 nor FOXK2 binding was found at the promoter sites of *CMTM7* and *SOCS3*, which were thus included as negative controls for further characterization (Figs. 4F and S9D). In agreement with ChIP-seq data, chromatin immunoprecipitation and quantitative PCR (ChIP-qPCR) assay in *ASXL1*^+/N590^ and *ASXL1*^+/−^ cells confirmed endogenous FOXK1 bindings at the promoter sites of *TXNIP*, *VHL*, *SOCS1*, and *SOCS2*, but not *CMTM7* (Fig. S9E). Moreover, BAP1 bindings at the promoter sites of FOXK1/K2 direct target genes (e.g., *TXNIP*, *VHL*, *SOCS1*, *SOCS2*, *MAGI1*, and *ZNF516*) were readily detected, and interestingly, single knockdown of either *FOXK1* or *FOXK2* moderately decreased the BAP1 occupancy, and double knockdown of both *FOXK1* and *FOXK2* dramatically decreased BAP1 bindings at the promoters of these genes (Fig. 4B–E, S9F, S9B and S9C). As a result, the H2AK119 mono-ubiquitination levels at the corresponding promoter regions were modestly increased by single knockdown of either *FOXK1* or *FOXK2*, and dramatically increased by double knockdown of both *FOXK1* and *FOXK2* (Fig. 4B–E, S9B and S9C).

More importantly, we found that BAP1 bindings at the promoters of *TXNIP*, *VHL*, *SOCS1*/*2*, *MAGI1*, and *ZNF516* were significantly increased in *ASXL1*^+/−^ cells compared to *ASXL1*^+/N590^ cells (Fig. 4B–E, S9B and S9C). Consequently, the H2AK119 mono-ubiquitination levels at the corresponding promoter regions of these FOXK1/K2 target genes were significantly decreased (Fig. 4B–E, S9B and S9C). Although ASXL1 is known to play a role in maintaining H3K27me3 levels by interacting with PRC2 complex, we found that the H3K27me3 levels at the FOXK1/K2 target gene promoters were comparable between *ASXL1*^+/−^ and *ASXL1*^+/N590^ cells (Fig. S10), excluding the involvement of PRC2 and re-affirming that the expression of C-terminally truncated mutant ASXL1 proteins dominantly inhibits the function of wild-type ASXL1 at least in part by suppressing the recruitment of BAP1 to the promoters of FOXK1/K2 target genes and dysregulating gene expression through H2AK119 mono-ubiquitination.

### Tumor-derived ASXL1 mutant regulates glucose metabolism and HIF-1α and STAT3 signaling pathways

**Figure 5:**
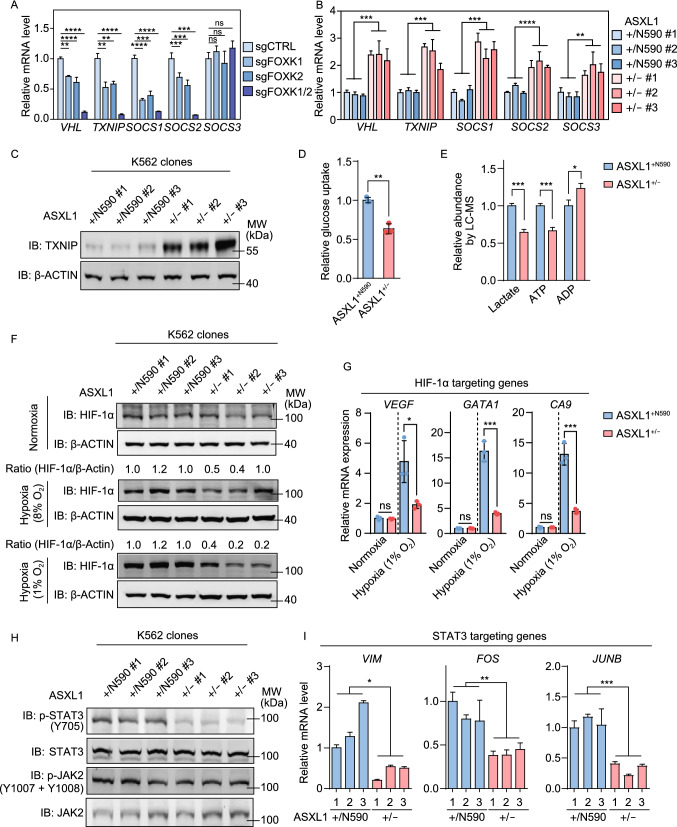
**Tumor-derived ASXL1 mutant regulates glucose metabolism, oxygen sensing, and JAK-STAT3 signaling pathways**. (A) The mRNA expression of indicated genes in *FOXK1* and/or *FOXK2* knockout K562 cell pools, as determined by qRT-PCR. (B) The mRNA expression of indicated genes in *ASXL1*^+/N590^ and *ASXL1*^+/−^ K562 clones, as determined by qRT-PCR. (C) TXNIP protein level in *ASXL1*^+/N590^ and *ASXL1*^+/−^ clones, as determined by Western blot. (D) Glucose uptake in *ASXL1*^+/N590^ and *ASXL1*^+/−^ clones, as measured using a Glucose Assay Kit as described in the “MATERIALS AND METHODS” section. (E) Relative concentration of lactate, ATP, and ADP in *ASXL1*^+/N590^ and *ASXL1*^+/−^ K562 clones, as measured by LC–MS/MS as described in the “MATERIALS AND METHODS” section. (F) The protein levels of HIF-1α in *ASXL1*^+/N590^ and *ASXL1*^+/−^ K562 clones under the culture conditions of normoxia or hypoxia (1% and 8% O_2_, exposure for 48 h)*.* (G) The mRNA expression of HIF-1α target genes in *ASXL1*^+/N590^ and *ASXL1*^+/−^ K562 clones under the culture conditions of normoxia or hypoxia (1% O_2_, exposure for 48 h). (H) JAK2, STAT3, and their site-specific phosphorylation levels in *ASXL1*^+/N590^ and *ASXL1*^+/−^ clones. (I) The mRNA expression of STAT3 target genes in *ASXL1*^+/N590^ and *ASXL1*^+/−^ clones, as determined by qRT-PCR. Asterisks denote statistical significance with two-tailed Student’s *t*-test (B, D, E, G, I) or one-way ANOVA (A). *P < 0.05; **P < 0.01; ***P < 0.001; ****P < 0.0001 for the indicated comparison; ns = not significant

Consistent with H2AK119 mono-ubiquitination mediated by PRC1 as a prevalent repressive histone modification, the expression of *TXNIP*, *VHL*, *SOCS1*/*2*, *MAGI1*, and *ZNF516* genes was down-regulated by double knockdown of both *FOXK1* and *FOXK2* in K562 cells (Figs. 5A and S11A) and by the presence of the mutant *ASXL1* allele (Figs. 5B and S11B). The mRNA expression of *CMTM7* gene was down-regulated by mutant ASXL1, but failed to be regulated by knockdown of *FOXK1* and/or *FOXK2* (Fig. S11A and S11B), suggesting that *CMTM7* is regulated by mutant ASXL1 in a FOXK1/K2-independent manner.

TXNIP is a potent negative regulator of glucose uptake and aerobic glycolysis (Waldhart et al., 2017), and its mRNA level is commonly reduced in diverse tumor types (Nishizawa et al., 2011). In agreement with the decreased mRNA expression of *TXNIP*, protein level of TXNIP was also remarkably reduced by depletion of FOXK1/K2 (Fig. S12A) or C-terminally truncated ASXL1 mutant (Fig. 5C). Supporting the inhibitory role of TXNIP in glucose homeostasis, knockdown of *TXNIP* in K562 cells by two different sgRNAs led to enhanced glucose uptake by more than 1.5-fold (*P* < 0.01) (Fig. S12B and S12C). In *ASXL1*^+/N590^ cells, downregulation of TXNIP was associated with significantly (*P* < 0.01) increased glucose uptake (Fig. 5D) and higher intracellular levels of lactate and ATP (Fig. 5E) as compared to *ASXL1*^+/−^ cells.

*VHL* is a component of an E3 ubiquitin ligase complex which targets hypoxia-inducible factor-1 alpha (HIF-1α) for ubiquitination and proteasomal degradation under hypoxia condition. We found that *VHL* mRNA expression was significantly down-regulated by C-terminally truncated ASXL1 mutant (Fig. 5B), which encouraged us to check HIF-1α protein. No change in HIF-1α protein levels was observed between *ASXL1*^+/N590^ and *ASXL1*^+/−^ K562 cells under normoxic culture conditions (21% O_2_). HIF-1α protein level was, however, remarkably higher in *ASXL1*^+/N590^ than *ASXL1*^+/−^ cells under hypoxic conditions (8% or 1% O_2_) (Fig. 5F). As a result, the mRNA expression of HIF-1α direct target genes was upregulated in *ASXL1*^+/N590^ cells compared to *ASXL1*^+/−^ cells under hypoxia (1% O_2_), including vascular endothelial growth factor (VEGF), GATA binding protein 1 (GATA1), and carbonic anhydrase IX (CA9) (Fig. 5G).

Aberrant activation of JAK-STAT3 signaling has been seen in diverse types of cancer including myeloid neoplasm (Thomas et al., 2015). Supporting the inhibitory effects of SOCS1 and SOCS2 on JAK signaling, we found down-regulation of *SOCS1*/2 by mutant ASXL1 was associated with increased STAT3 Y705 phosphorylation, an indicator of STAT3 activation, in *ASXL1*^+/N590^ as compared to *ASXL1*^+/−^ cells (Fig. 5H). And higher STAT3 Y705 phosphorylation was associated with up-regulation of STAT3 target gene expression in *ASXL1*^+/N590^ cells, including Vimentin (*VIM*), *FOS*, and *JUNB* (Fig. 5I).

Collectively, these results suggest that the expression of C-terminally truncated ASXL1 mutant impedes BAP1-ASXL1-FOXK1/K2 pathway to down-regulate *TXNIP*, *VHL*, and *SOCS1*/*2* and thus promotes glucose uptake for energy production and enhances oncogenic HIF-1α and JAK/STAT3 signaling pathways.

### Tumor-derived ASXL1 mutants promote leukemia cell growth through increased cell cycle progression and decreased apoptosis

**Figure 6:**
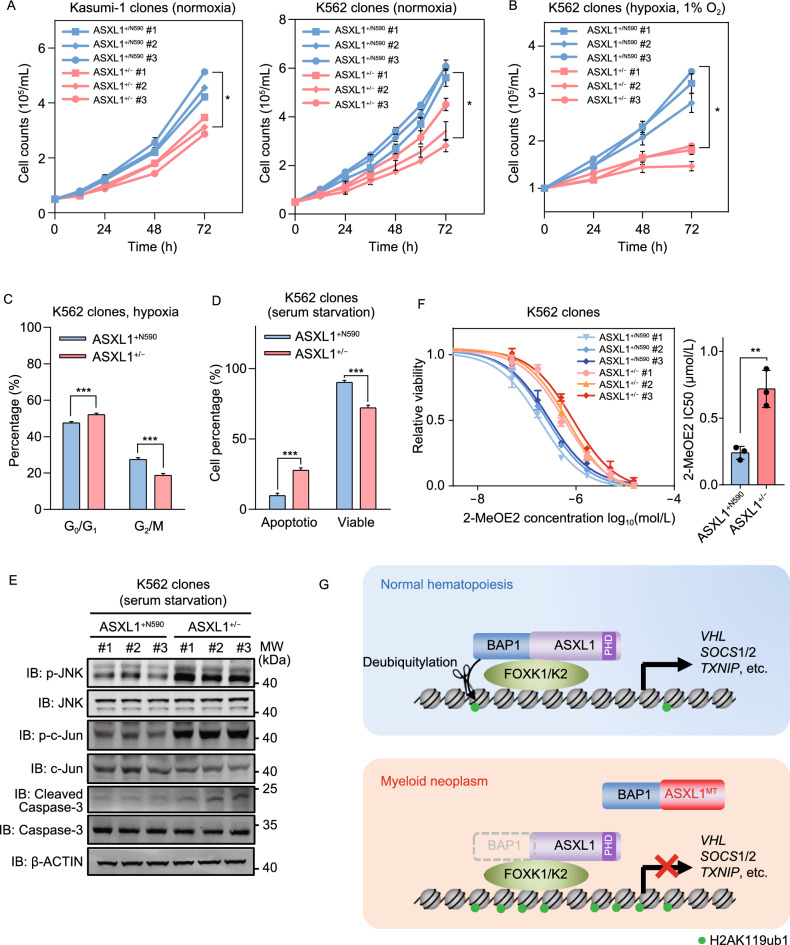
**Tumor-derived ASXL1 mutants promote leukemia cell growth through increased cell cycle progression and decreased apoptosis**. (A and B) Cell proliferation of *ASXL1*^+/N590^ and *ASXL1*^+/−^ K562 clones, as well as *ASXL1*^+/N646^ and *ASXL1*^+/−^ Kasumi-1 clones under normoxia or hypoxia (1% O_2_) condition for the indicated time periods. (C) The ratios of G0/G1-phase and G2/M-phase in *ASXL1*^+/N590^ and *ASXL1*^+/−^ clones under hypoxia (1% O_2,_ 24 h). (D) Cell apoptosis and viability in *ASXL1*^+/N590^ and *ASXL1*^+/−^ clones upon serum starvation. The indicated K562 cells were maintained in RPMI-1640 medium without FBS for 48 h. (E) Apoptosis-related markers in *ASXL1*^+/N590^ and *ASXL1*^+/−^ clones upon serum starvation. Cells were cultured as mentioned above in (D). (F) *ASXL1*^+/N590^ and *ASXL1*^+/−^ K562 clones were treated with 2-MeOE2 (HIF-1α inhibitor) for 24 h, and the cytotoxicity was determined by CCK-8 assay as described in the “MATERIALS AND METHODS” section. (G) Working model. C-terminally truncated ASXL1 mutant is expressed at a much higher protein level than wild-type ASXL1, and loses interaction with transcription factors such as FOXK1 and FOXK2, but still interacts with BAP1. Thus, the mutant ASXL1 protein inhibits the interaction between BAP1 and wild-type ASXL1 in a dominant-negative manner, reduces BAP1 enrichment and increases H2AK119 mono-ubiquitination at the promoters of FOXK1/K2 target genes, and impairs the function of BAP1-ASXL1-FOXK1/K2 axis to regulate target genes and leukemia cell growth. Asterisks denote statistical significance with two-tailed Student’s *t*-test (A, B, C, D, F). *P < 0.05; ***P < 0.001 for the indicated comparison; ns = not significant

Finally, we examined the functional consequence of deleting the C-terminally truncated ASXL1 mutant on leukemia cell growth. We found that deletion of mutant *ASXL1* allele significantly decreased cell proliferation by as much as 32% (*P* = 0.01) and 40% (*P* = 0.01) in Kasumi-1 and K562 cells, respectively (Fig. 6A). And the difference became even more pronounced under hypoxia condition, as *ASXL1*^+/−^ K562 cells poorly proliferated under 1% O_2_ culture condition, while *ASXL1*^+/N590^ cells still exhibited rapid proliferation. As a result, deletion of mutant *ASXL1* allele decreased cell proliferation by as much as 45% (*P* < 0.01) in K562 under hypoxia condition (Fig. 6B). Moreover, deletion of mutant *ASXL1* allele changed cell cycle and decreased the G2/M cell population by 22% (*P* < 0.05) and 25% (*P* < 0.001) under normoxic and hypoxic conditions, respectively (Figs. S13A, S13B and 6C). Deletion of the mutant *ASXL1* allele also significantly (*P* < 0.001) increased apoptosis and decreased cell viability upon serum deprivation (Fig. 6D). The observed increase in cell apoptosis in *ASXL1*^+/−^ K562 cells upon serum starvation was associated with activation of the c-Jun NH_2_-terminal kinase (JNK) pathway, one of the major apoptosis-related signaling cassettes, as evidenced by higher phosphorylation levels of JNK and its downstream target c-Jun in *ASXL1*^+/−^ than *ASXL1*^+/N590^ cells (Fig. 6E). In accord, cleaved Caspase-3, an apoptotic marker, was increased in *ASXL1*^+/−^ compared to *ASXL1*^+/N590^ cells upon serum deprivation (Fig. 6E). Furthermore, *ASXL1*^+/N590^ cells displayed enhanced clonogenicity as compared to *ASXL1*^+/−^ cells (Fig. S13C).

Supporting the tumor suppressive effects of *TXNIP* and *MAGI1*, we found that knockdown of *TXNIP* or *MAGI1* in *ASXL1*^+/−^ K562 cells led to increased proliferation (Fig. S12D and S12G) and higher cell viability under serum deprivation (Fig. S12E and S12H). These findings indicate that C-terminally truncated ASXL1 mutant promotes cell cycle progression and attenuate apoptosis at least in part by repressing the above-mentioned tumor suppressor genes, especially under stress conditions such as hypoxia and serum deprivation.

Numerous studies have established the oncogenic roles of VHL/HIF-1α and SOCS/JAK-STAT pathways in regulating leukemia cell growth and hematological malignancies (Labno-Kirszniok et al., 2013; Moura et al., 2018). We found that C-terminally truncated ASXL1 mutant mildly increased the cellular susceptibility to STAT3 inhibitor, with the IC_50_ for C188-9 being 7.9 ± 0.72 μmol/L and 11.84 ± 1.31 μmol/L in *ASXL1*^+/N590^ and *ASXL1*^+/−^ cells, respectively (Fig. S14). Interestingly, the IC_50_ for 2-methoxyestradiol (2-MeOE2, a HIF-1α inhibitor) was 0.25 ± 0.04 μmol/L and 0.72 ± 0.11 μmol/L in *ASXL1*^+/N590^ and *ASXL1*^+/−^ cells, respectively (Fig. 6F), indicating that *ASXL1*-mutated cells are more sensitive to HIF-1α inhibitor.

## DISCUSSION

Mutations in ASXL1 are associated with poor prognosis across the spectrum of malignant myeloid diseases (Gelsi-Boyer et al., 2012; Micol and Abdel-Wahab, 2016), and nearly all ASXL1 mutations lead to the production of C-terminally truncated mutant ASXL1 proteins. To study the oncogenic roles of C-terminally truncated ASXL1, previous studies have employed either transgenic expression (Inoue et al., 2013; Yang et al., 2018) or knock-in mouse models (Hsu et al., 2017; Nagase et al. 2018). It has to be noted that transgenic expression is an overexpression system, and knock-in mouse models investigate the effect of Asxl1 mutation in the absence of other collaborative leukemic mutations. In this study, we explore the molecular and cellular effects of genetic deletion of mutant *ASXL1* allele in the context of other collaborating oncogenic alterations in leukemia cell lines. Using the isogenic *ASXL1*^+/*MUT*^ and *ASXL1*^+/−^cells, we confirm previous findings (Balasubramani et al., 2015; Inoue et al., 2016; Asada et al., 2018) and show that mutant ASXL1 promotes the deubiquitylase activity of BAP1 to decrease global H2AK119 mono-ubiquitination. In contrast, we uncover that mutant ASXL1 increases H2AK119 mono-ubiquitination at specific genomic loci. Mechanistically, C-terminally truncated *ASXL1* mutants retain BAP1 bindings but lose interaction with FOXK1 and FOXK2. As the result of their high expression, the neomorphic ASXL mutants sequester BAP1 from wild-type ASXL1 and impair the function of ASXL1-BAP1-FOXK1/K2 axis in regulating target genes (Fig. 6G).

We show here that a significant portion of mutant ASXL1’s function is linked to FOXK1 and FOXK2, as more than 60% of genes differentially regulated by C-terminal truncation mutant ASXL1 are direct targets of FOXK1/K2. Although both transcriptional activation (Sukonina et al., 2019) and repression (Sun et al., 2016; Li et al., 2019) can be regulated by FOXK1 and FOXK2, our study supports the overlapping and transcriptional activation role of FOXK1/K2 and is consistent with the gene silencing role of H2AK119 mono-ubiquitination mediated by the PR-DUB complex. Supporting this notion, knockdown of FOXK1/K2 or deletion of mutant *ASXL1* allele leads to decreased H2AK119 mono-ubiquitination at the promoter regions of FOXK1/K2 target genes (Fig. 4). Among the dysregulated FOXK1/K2 targets by mutant ASXL1 are multiple tumor suppressors: *VHL* mutations and promoter hypermethylation play a crucial role in the development of AML (Labno-Kirszniok et al., 2013; Gossage et al., 2015; Moura et al., 2018); *SOCS1* and *SOCS2* are associated with dysregulation of cell growth, cancer-associated inflammation, and cell death induced by multiple cytokines and hormones (Duncan et al., 2017; Jiang et al., 2017); *TXNIP* plays an important role in glucose uptake and redox homeostasis (Waldhart et al., 2017). Phenotypically, mutant ASXL1 impairs the BAP1-ASXL1-FOXK1/K2 transcriptional nexus and downregulates TXNIP, VHL, and SOCS1/2, and consequently promotes glucose metabolism and enhances oncogenic HIF-1α and JAK-STAT3 signaling pathways known to be altered in hematological malignancies.

In addition to FOXK1/K2, we discover abundant association of ASXL1 with HCF-1 which is known to interact with BAP1 (Machida et al., 2009; Yu et al., 2010), as well as 25 other DNA-binding transcription regulators that have not been previously implicated in ASXL1 interaction. Among them, BCL2 associated transcription factor 1 (BCLAF1) encodes a death-promoting transcriptional repressor and can regulate the differentiation of normal hematopoietic progenitors and is deregulated in AML (Dell'Aversana et al., 2017). Nucleophosmin 1 (NPM1) is frequently mutated in AML and exerts multifunctions in the regulation of centrosome duplication, ribosome biogenesis, genomic stability, histone chaperone function, and transcription (Verhaak et al., 2005; Heath et al., 2017); GATA binding protein 1 (GATA1) is a DNA-sequence specific TF which regulates erythroid development by controlling the fetal-to-adult hemoglobin switch, and correlates with the degree of myeloid phenotypic changes in Npm1/Flt3-ITD mice (Sportoletti et al., 2019). The results presented here suggest a previously unrecognized mechanism by which ASXL1 forms partnerships with many DNA-binding transcription regulators including TFs and guides the BAP1-containing PR-DUB complex to specific loci in the genome. Together with the finding that ASXL1^N646^ retains BAP1 binding but loses interaction with at least nine additional DNA-binding transcription regulators beside FOXK1/K2, it raises a possibility that the interference with BAP1-ASXL1-TF axis may represent a mechanism for C-terminally truncated mutant ASXL1 to regulate target genes and leukemia cell growth.

## MATERIALS AND METHODS

### Antibodies

All the antibodies used in the study were purchased commercially, including Anti-Flag tag (Sigma, catalog F7425), anti-Myc (HuaBio, catalog EM31105), anti-BAP1 (Santa Cruz Biotechnology, catalog sc-28383 for IP and WB; GST, catalog 78105 for ChIP), anti-ASXL1 (Abcam, catalog ab228009; Abcam, catalog ab221455), anti-FOXK1 (Abcam, catalog ab18196), anti-FOXK2 (Abcam, ab5298), anti-HCF-1 (GST, catalog 50708), anti-H2AK119 mono-ubiquitination (Abcam, catalog ab193203), anti-H3K27me3 (Abcam, catalog ab6002), anti-Histone H2A (Abcam, catalog ab18255), anti-Histone H3 (CST, catalog 9715), anti-TXNIP (Abcam, catalog ab188865), anti-HIF-1α (Abcam, catalog ab216842), anti-STAT3 (CST, catalog 12640), anti-p-STAT3 (CST, catalog 9145), anti-JAK2 (Abcam, catalog ab108596), anti-p-JAK2 (Abcam, catalog ab32101), anti-GAL4 (Santa Cruz Biotechnology, catalog sc-510), anti-ACTIN (Genscript, catalog A00702).

### Plasmids

For co-immunoprecipitation assay, cDNAs encoding full-length human ASXL1, BAP1, FOXK1 and truncated ASXL1 were cloned into Flag or Myc-tagged vectors (pRK7-N-Flag, pRK7-N-Myc); For depleting ASXL1, MAGI1 and BAP1, *shASXL1*, sh*BAP1* and *shMAGI1* were cloned into PLKO.1 vector; For depleting ASXL1, FOXK1, FOXK2 and TXNIP in K562 cells, *sgASXL1*, *sgFOXK1*, *sgFOXK2* and *sgTXNIP* were cloned into Plenti-Crispr vector. All constructs have been verified by DNA sequencing.

For *in vitro* pull-down assay, the plasmid overexpressing ASXL1 (residues 450–1,300) was constructed by cloning cDNAs encoding SBP and ASXL^450–1,300^ into pRK7-N-Flag vector, and the plasmid overexpressing Flag-tagged FOXK1 was constructed by cloning cDNAs encoding full-length FOXK1 into pRK7-N-Flag vector.

### Cell culture

HEK293T cells (CRL-11268, ATCC, Manassas, VA, USA) were cultured in DMEM containing 10% fetal bovine serum (FBS, GBICO), penicillin, and streptomycin; U2OS, K562 (CCL-243, ATCC) and Kasumi-1 (CRL-2724, ATCC) cells were cultured in RPMI-1640 medium supplemented with 10% FBS, penicillin, and streptomycin.

Hypoxic incubation was performed in a water-jacketed, humidified, multigas tissue culture incubator equipped with nitrogen and carbon dioxide. Parallel normoxic incubation was carried out in standard humidified 5% carbon dioxide tissue culture incubators.

### *In vitro* pull-down assay

SBP-tagged ASXL1^450–1,300^ was overexpressed in HEK293T cells and immobilized to Streptavidin beads. Flag-tagged full-length FOXK1 protein was overexpressed in HEK293T cells, purified by immunoprecipitation and eluted by Flag peptide. Immobilized ASXL1 protein was incubated with FOXK1 protein at 4 °C for 3 h and then washed for 3 times, followed by SDS-PAGE and Commassie blue staining.

### Mammalian two-hybrid screen

Human full-length ASXL1 CDS fused to VP16 transactivation domain (AD), Preys fused to Gal4 DNA-binding domain (DBD), UAS-Luciferase reporter plasmid, and CMV-Renilla control plasmid were co-transfected in HEK293T cells. At 48 h after transfection, the luciferase reporter activity was measured by a commercial kit (Promega E1910) using a Turner BioSystems Luminometer Reader (Promega). When the ASXL1-VP16 (AD) exists, the luciferase value is labeled as L1, and the Renilla value is labeled as R1. When the ASXL1-VP16 (AD) does not exist, the luciferase value is labeled as L2, and the Renilla value is labeled as R2. The relative luciferase activation is calculated as following: (L1/R1)/(L2/R2).

### Stable cells generation

For generation of stable K562 cell pools with *FOXK1* or *FOXK2* knockdown, sgRNAs targeting FOXK1 or FOXK2 in pLenti-crispr vector were used. Lentivirus was harvested after 24 h post transfection, and mixed with 8 μg/mL polybrene. K562 cells were infected for twice and selected in 1 μg/mL puromycin for 5 days.

For generation of *ASXL1*^+*/−*^ monoclonal cells, sgRNAs against ASXL1 in pLenti-Crispr vector were transfected in K562 and Kasumi-1 cells. Lentivirus was harvested after 24 h post transfection, and mixed with 8 μg/mL polybrene. K562 and Kasumi-1 cells were infected with lentivirus and then selected in 1 mg/mL puromycin for 5 days. Flow cytometry (Beckman) was applied to sort monoclonal cells into 96-well plates, following verification of ASXL1 deletion by Western blot.

### Cellular susceptibility to chemical inhibitors

K562 cells were seeded into 96-well flat plates (1.5 × 10^4^ cells per well) and then treated with increased concentrations of C188-9 or 2-MeOE2 at 37 °C for 24 h. Cell viability was assessed using a Cell Counting Kit-8 (CCK-8; Beyotime Biotechnology, Inc., Shanghai, China), according to the manufacturer’s instructions. The optical density (OD) was measured at 450 nm using a fluorescence spectrofluorometer (F-7000; Hitachi High-Technologies Corporation, Tokyo, Japan), and the reference OD was subtracted. RPMI-1640 containing 10% FBS with 0.02% DMSO served as the reference group. Each experiment was performed in triplicate and repeated three times.

### Transfection and immunoprecipitation (IP)

Plasmids were transfected into cells using polyethylenimine (PEI) or Lipofectamine 2000 (Invitrogen) following the manufacturer’s instruction. Cells were washed with cold phosphate buffered saline (PBS) once and lysed in lysis buffer (50 mmol/L Tris–HCl, 150 mmol/L NaCl, 0.3% sodium deoxycholate and 0.5% NP-40, pH 7.4). For IP, cell lysates were incubated with anti-Flag beads (Sigma-Aldrich, catalog F1804, clone M2, 1:200) or anti-BAP1 antibody for 3 h at 4 °C.

### Immunofluorescence assay

Cells were washed with cold PBS and fixed with 4% Formaldehyde (Sangon) for 15 min at room temperature. Then, cells were treated with 0.3% Triton X-100 for cell perforation at room temperature for 15 min, and were incubated with blocking buffer (3% BSA in PBS) for 1 h, followed by incubation at 4 °C overnight with the primary antibody against Flag, and Alex Fluor 594 (Green) conjugated secondary antibody (Invitrogen) at room temperature for 1 h. Cell nucleus was stained with DAPI (Invitrogen). The cells were examined by fluorescence microscopy (Olympus America Inc, Center Valley, PA).

### Immunoprecipitation-mass spectrometry (IP-MS)

Whole cell lysates extract from U2OS cells expressing Flag-tagged full-length or truncated ASXL1 protein was incubated with a 300 µL packed volume of anti-Flag-beads (M2-agarose, Sigma) overnight at 4 °C with rotation. The beads were collected by centrifugation at 700 × *g* for 5 min and washed for 6 times with wash buffer (20 mmol/L Tris–HCl, pH 8.0, 300 mmol/L NaCl, 1.5 mmol/L MgCl_2_, 0.2 mmol/L EDTA, 10% glycerol, 0.2 mmol/L PMSF, 1 mmol/L DTT, 1 µg/mL aprotinin and 1 µg/mL leupeptin). The samples were boiled in SDS loading buffer and run shortly on SDS-PAGE gel. A gel slice containing the purified proteins was isolated for MS analysis. Briefly, the MS analysis was performed by using a standard proteomics workflow. The proteins were in-gel digested using 10 mmol/L DTT (45 min at 56 °C), followed by 40 mmol/L iodoacetamide (30 min at room temperature in dark) and overnight incubation with trypsin (enzyme: sample ratio ~ 1:20, pH 8.0, 37 °C). Separation and MS detection were achieved by using nano liquid chromatography coupled online to tandem MS using a data dependent acquisition method.

### Gene expression profiling by RNA sequencing

RNA-seq transcriptome library was prepared following TruSeq™ RNA sample preparation Kit from Illumina (San Diego, CA) using 5 μg of total RNA. Secondly double-stranded cDNA was synthesized using a SuperScript double-stranded cDNA synthesis kit (Invitrogen, CA) with random hexamer primers (Illumina). Then the synthesized cDNA was subjected to end-repair, phosphorylation and ‘A’ base addition according to Illumina’s library construction protocol. Libraries were size selected for cDNA target fragments of 200–300 bp on 2% Low Range Ultra Agarose followed by PCR amplified using Phusion DNA polymerase (NEB) for 15 PCR cycles. After quantified by TBS380, paired-end RNA-seq sequencing library was sequenced with the Illumina HiSeq 4000 (2 × 150 bp read length). The raw paired end reads were trimmed and quality controlled by SeqPrep (https://github.com/jstjohn/SeqPrep) and Sickle (https://github.com/najoshi/sickle) with default parameters. Then clean reads were separately aligned to reference genome with orientation mode using TopHat (https://tophat.cbcb.umd.edu/, version 2.0.0) (Trapnell et al. 2009) software. The mapping criteria of bowtie was as follows: sequencing reads should be uniquely matched to the genome allowing up to 2 mismatches, without insertions or deletions. Then the region of gene was expanded following depths of sites and the operon was obtained. In addition, the whole genome was split into multiple 15 kbp windows that share 5 kbp. New transcribed regions were defined as more than 2 consecutive windows without overlapped region of gene, where at least 2 reads mapped per window in the same orientation.

### RNA isolation and qRT-PCR analysis

Total RNA was extracted from cultured cells by Trizol reagent (Invitrogen) following the manufacturer’s instruction. RNA was reversely transcribed with oligo-dT primers. Diluted cDNA was then used for real-time PCR with gene-specific primers in the presence of TB Green™ Advantage® qPCR Premix (Takara) by QuantStudio 6 Flex Real-Time PCR system (Applied Biosystems). β-ACTIN was used as a housekeeping control. Primer sequences were listed in the Table S5.

### Chromatin immunoprecipitation (ChIP)-qPCR assays

ChIP-qPCR assays were performed as described previously (Lan et al. 2007). Briefly, cells were cross-linked with 1% paraformaldehyde. After sonication at 4 °C for 20 min (Bioruptor, low mode), chromatin was immunoprecipitated at 4 °C for 3 h with the antibody against BAP1, H2AK119 mono-ubiquitination and H3K27me3 or rabbit IgG. Antibody-chromatin complexes were pulled-down using protein A-Sepharose (RepliGen), and then washed and eluted by elution buffer. After cross-link reversal and proteinase K (Takara) treatment, immunoprecipitated DNA was extracted with PCR Purification Kit (QIAGEN). The DNA fragments were further analyzed by real-time quantitative PCR using the primers as listed in Table S5.

### Glucose uptake assay

Glucose uptake was quantified from culture medium using Glucose (GO) Assay Kit (Sigma, catalog GAGO20). Assays were performed according to the manufacturer's instructions.

### Intracellular metabolite profiling

Cells were quickly washed twice with PBS prepared in LC/MS grade water and fixed by immediate addition of 1 mL 80% (*v*/*v*) pre-chilled (− 80 °C) methanol into culture plates. Metabolites were extracted by rotating at 4 °C for 1 h. Samples were then spun down at 17,000 × *g* for 10 min at 4 °C and the supernatant was collected. Cell extracts were analyzed by ultrahigh performance liquid chromatograph (Acquity, Waters) coupled to a Q Exactive hybrid quadrupole-orbitrap mass spectrometer (Thermo Fisher).

### Cell proliferation assay

Cells were seeded in 6-well plates at 10^5^ cells per well and incubated at 37 °C. The number of cells per well was determined at indicated time points by removing cells from triplicate wells, pelleting by centrifugation, resuspension in a small volume of medium, and counting of viable cells using trypan blue in a hemocytometer.

### Cell cycle assay

Cells were fixed in cold 70% ethanol for at least 2 h and then stained with propidium iodide (PI) (Cell Cycle and Apoptosis Analysis Kit; Shanghai Yeasen Biotechnology) at room temperature for 15 min. The cells were then counted using an Accuri™ C6 flow cytometry (BD Biosciences).

### Cell apoptosis assay

Cells were seeded in 6-well plates in RPMI-1640 medium at the density of 2 × 10^5^ cells per well. Subsequently, cells were collected and stained by FITC Apoptosis Detection Kit (BD) following the manufacturer’s instruction. The stained cells were detected by BD Accuri C6 to calculate the percentage of apoptotic cells (Annexin V-positive) and viable cells (both PI and Annexin V-negative).

### Colony forming unit assay

K562 cells were seeded in 12-well plate and cultured in MethoCult H4230 (Stem Cell Technologies Inc.) supplemented with 10% FBS. After 7 days the plates were scored for colony forming units (CFUs) according to manufacturer’s instruction. Colonies containing more than 20 cells were counted with a 4 × objective.

### Quantification and statistical analysis

Statistical analyses were performed with a two-tailed unpaired Student’s *t*-test. All data shown represent the results obtained from triplicated independent experiments with standard errors of the mean (mean ± SD). The values of *P* < 0.05 were considered statistically significant.

## Electronic supplementary material

Below is the link to the electronic supplementary material.

Supplementary file1 (PDF 1570 kb)

Supplementary file2 (XLSX 12 kb)

Supplementary file3 (XLSX 16013 kb)

Supplementary file4 (XLSX 27 kb)

Supplementary file5 (XLSX 49 kb)

Supplementary file6 (XLSX 11 kb)
