# Supplementary material for: Tumor-derived neomorphic mutations in ASXL1 impairs the BAP1-ASXL1-FOXK1/K2 transcription network
Source: Protein Cell. 2020 Jul 18;12(7):557–77. doi: 10.1007/s13238-020-00754-2 (PMC8225741; doi:10.1007/s13238-020-00754-2)
Supplement: Supplementary file 1 — Supplementary file1 (PDF 1570 kb) [file 13238_2020_754_MOESM1_ESM.pdf]

Supplemental Figures

Tumor-derived neomorphic mutations in ASXL1 impairs the BAP1-ASXL1-FOXK1/K2 transcription network

A **Y591X mutation in K562**

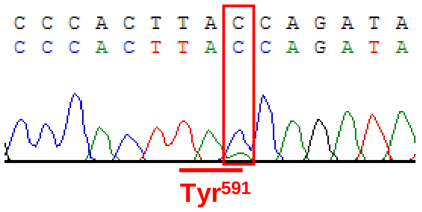

B **G646Wfs*12 mutation in Kasumi-1**

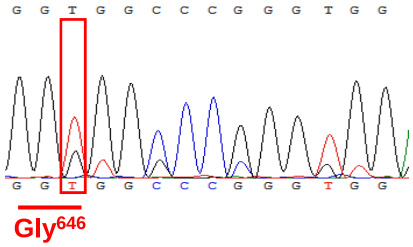

Figure S1. Verification of ASXL1 mutations in leukemia cell lines

Heterozygous Y591X mutation in K562 (A) and heterozygous G646Wfs*12 mutation in Kasumi-1 cells (B) were verified by PCR sequencing genomic DNA around the mutation sites.

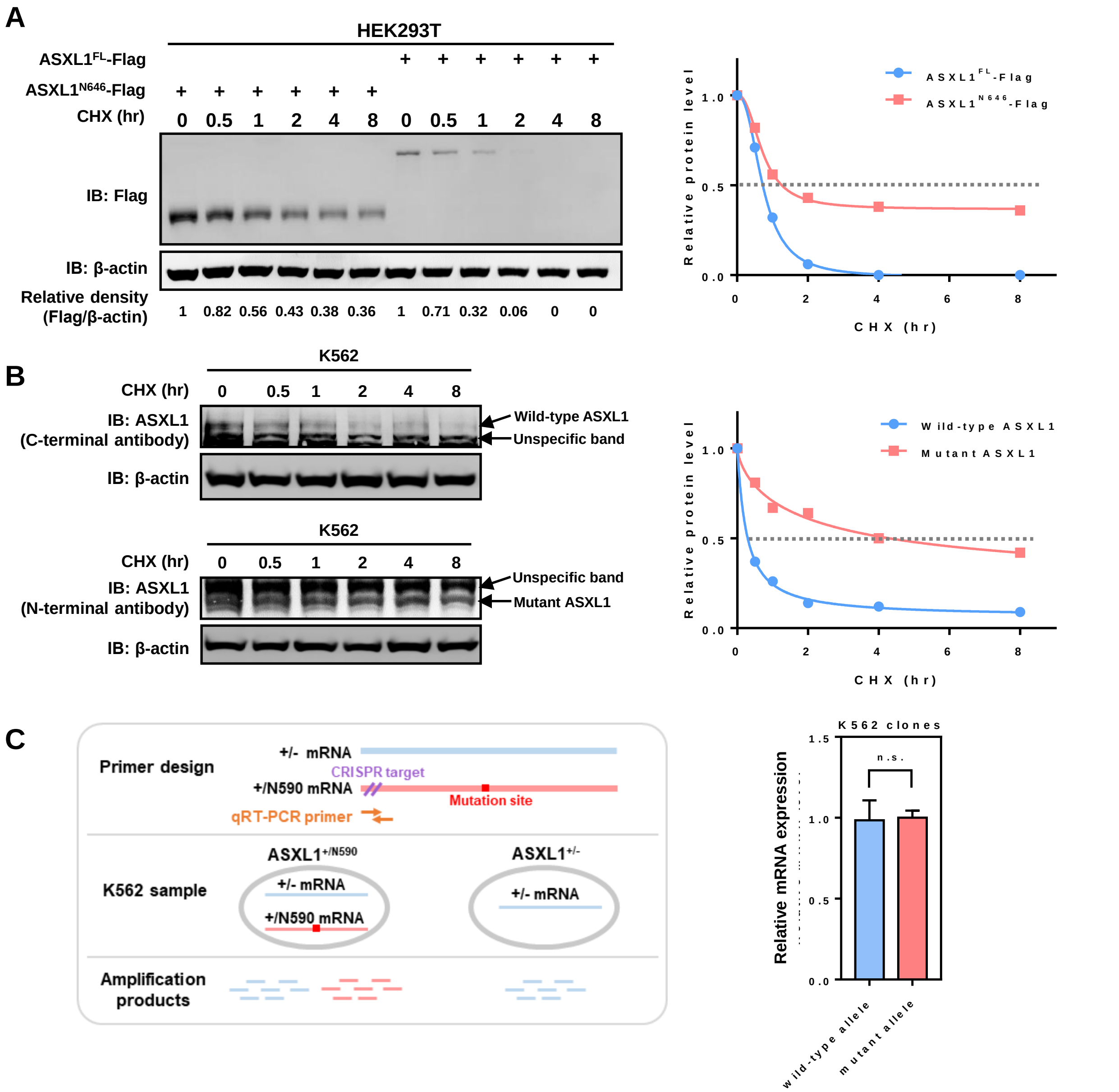

Figure S2. Protein stability of C-terminally truncated ASXL1 mutants is higher than wild-type ASXL1 protein

A, HEK293T cells overexpressing wild-type ASXL1or C-terminally truncated mutant were treated with 10 μg/ml of cycloheximide (CHX) to block protein synthesis before harvest. The levels of ectopically expressed ASXL1 proteins were detected by western blotting at the indicated time points (i.e. t=0, 0.5, 1, 2, 4, 8 hours after CHX treatment). The relative ASXL1 protein level was normalized with β-actin, and t=0 was defined as 1.

B, K562 cells were treated with CHX as described in (A), and the levels of endogenous wild-type and C-terminally truncated mutant ASXL1 were detected by western blotting. Two antibodies specific to the C-terminal or N-terminal sequence of ASXL1 protein were used. The relative ASXL1 protein level was normalized with β-actin, and t=0 was defined as 1.

C, The mRNA expression of endogenous wild-type and C-terminally truncated mutant ASXL1 in K562 cells was determined by qRT-PCR, and the detection process is shown (left). Relative mRNA level of ASXL1 mutant allele was calculated by subtracting ASXL1 mRNA level of ASXL1$^{+/-}$ K562 cells from ASXL1 mRNA level of ASXL1$^{+/N590}$ K562 cells.

Data information: Shown are average values with standard deviation (S.D.) of triplicated experiments.

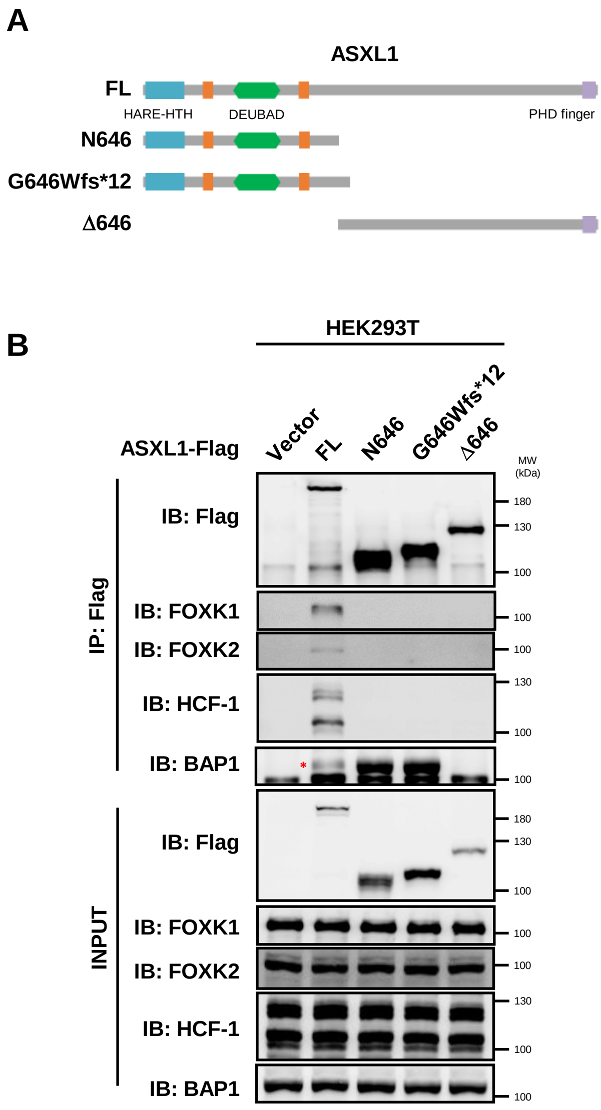

Figure S3. C-terminally truncated ASXL1 mutant fails to interact with FOXK1 and FOXK2

Flag-tagged ASXL1 truncations were overexpressed in HEK293T cells, following IP with anti-Flag beads and western blotting to detect the indicated endogenous proteins. The expected western blot band for BAP1 is marked with red star (*).

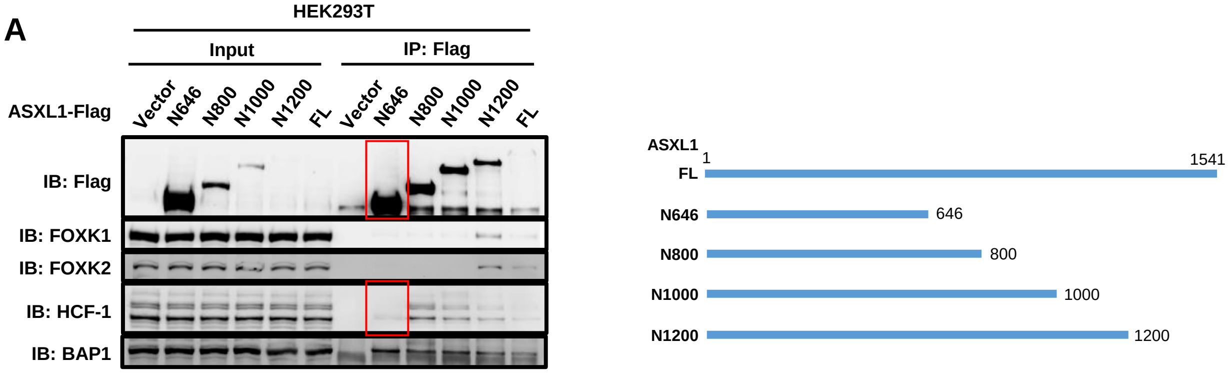

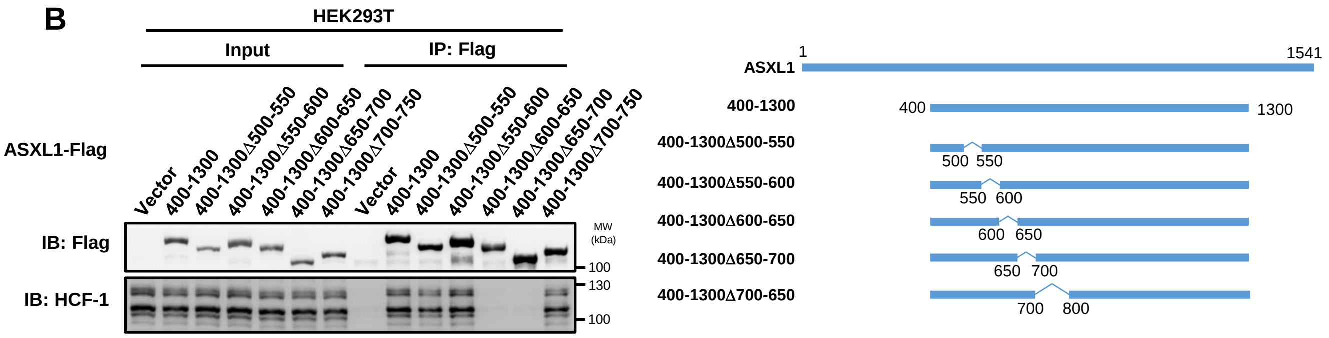

Figure S4. Mapping the domains in ASXL1 essential for HCF-1 interaction

A-B, Flag-tagged ASXL1 truncations were overexpressed HEK293T cells, and each truncation of ASXL1 was purified by IP with Flag beads, followed by western blot to detect interaction with endogenous HCF-1. As shown in (A), weak interaction with endogenous HCF-1 can be detected when Flag-ASXL1^{N646} is highly expressed (red squares).

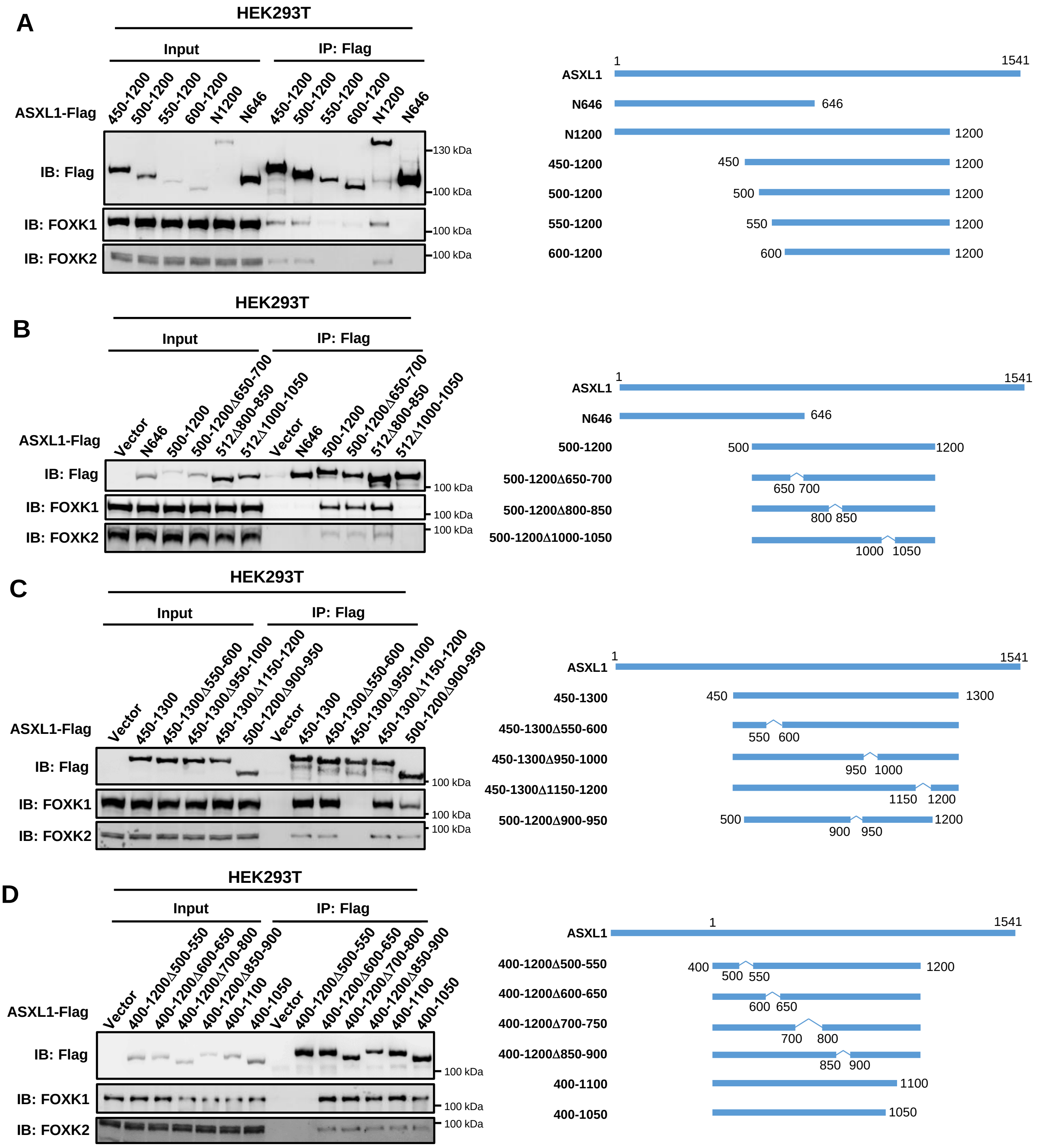

Figure S5. Mapping the domains in ASXL1 essential for FOXK1 and FOXK2 interaction

A-D, Flag-tagged ASXL1 truncations were overexpressed in HEK293T cells, and each truncation of ASXL1 was purified by IP with Flag beads, followed by western blot to detect its interaction with endogenous FOXK1 and FOXK2.

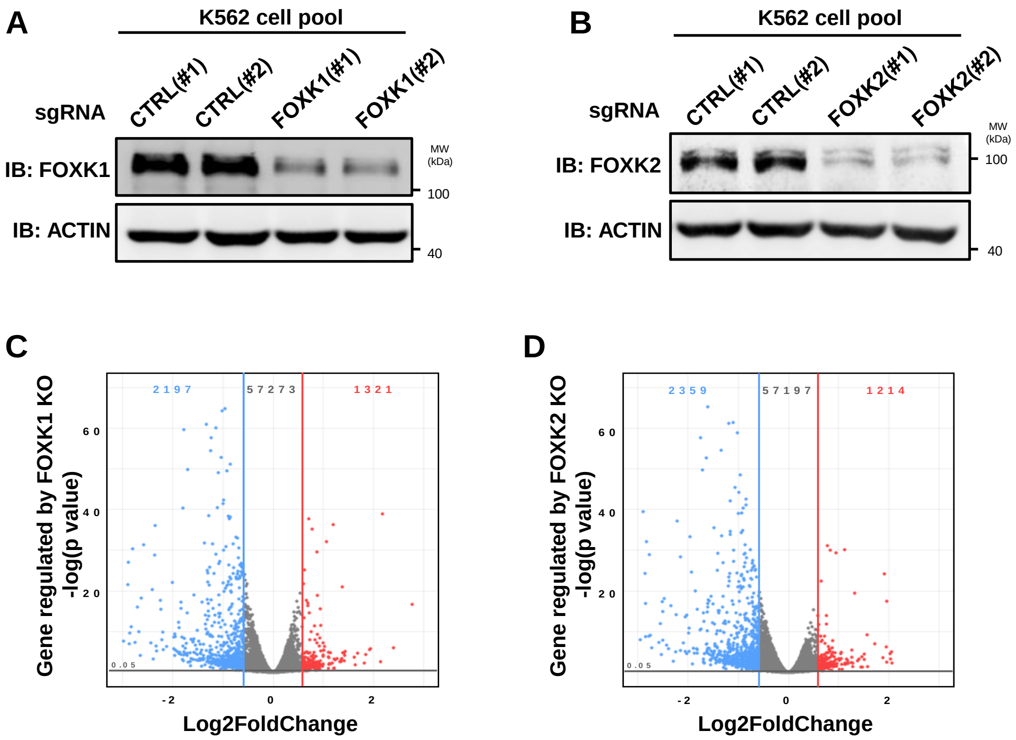

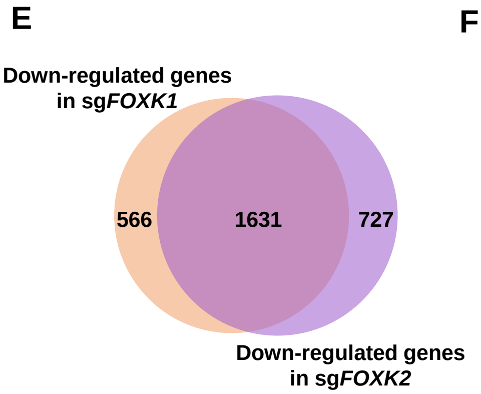

F

Term	Overlap	P-value	Genes
hematopoietic stem cell differentiation	2/7	0.0005	*ACE;HOXB4*
integrin activation	2/9	0.0009	*FN1;FERMT2*
positive regulation of stem cell differentiation	2/13	0.0019	*HOXB4;SOX6*
regulation of hormone biosynthetic process	1/8	0.0401	*STC2*
monoamine transport	1/7	0.0352	*SLC18A2*
regulation of transcription factor import into nucleus	2/20	0.0046	*BCL3;PIK3R2*
negative regulation of ion transmembrane transporter activity	2/22	0.0056	*TCAF2;HECW2*
ion homeostasis	1/8	0.0401	*KLHL3*
hematopoietic progenitor cell differentiation	2/23	0.0061	*ACE;HOXB4*
citrulline metabolic process	1/7	0.0352	*CPS1*

Figure S6. FOXK1 and FOXK2 are functionally overlapping in regulating gene expression

A-B, Verification of *FOXK1* (A) and *FOXK2* (B) knockdown efficiency in sg*FOXK1* and sg*FOXK2* K562 cell pools.

C-D, Volcano plots display differential expressed genes in sg*FOXK1* (C) and sg*FOXK2* (D) K562 cell pools.

E, A large proportion of genes down-regulated in sg*FOXK1* cells overlap with those which are down-regulated in sg*FOXK2* cells.

F, GO biological pathway analysis of overlapping genes down-regulated in sg*FOXK1* and sg*FOXK2* cells in K562.

A

Positive Prey	Relative Luciferase (normalized by FOXK2)	Interacts with ASXL1	Interacts with ASXL1N646
NPM1	3.8	Y	Y
MYCBP	3.7	Y	Y
HEY2	3.3	Y	Y
ING4	2.9	Y	Y
PSMC3	2.5	Y	Y
DDX17	2.3	Y	Y
ZNF317	2.0	Y	Y
XRCC6	1.9	Y	Y
NRBF2	1.8	Y	Y
NUP62	1.5	Y	Y
PA2G4	1.4	Y	Y
ZFP67	1.3	Y	Y
ELK3	1.3	Y	Y
RBBP4	1.2	Y	Y
GATAD2B	1.1	Y	Y
SND1	1.0	Y	Y

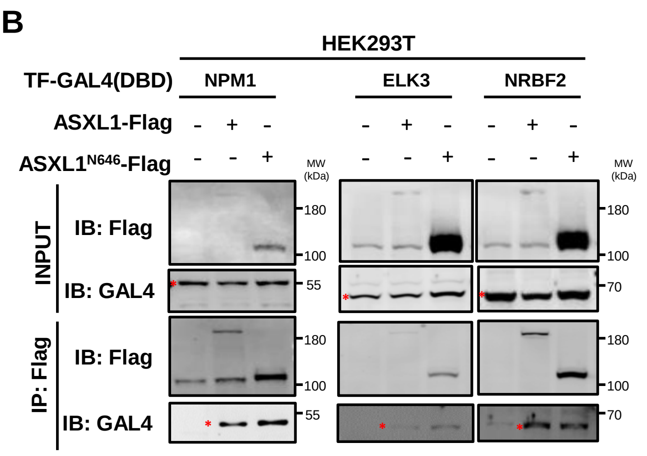

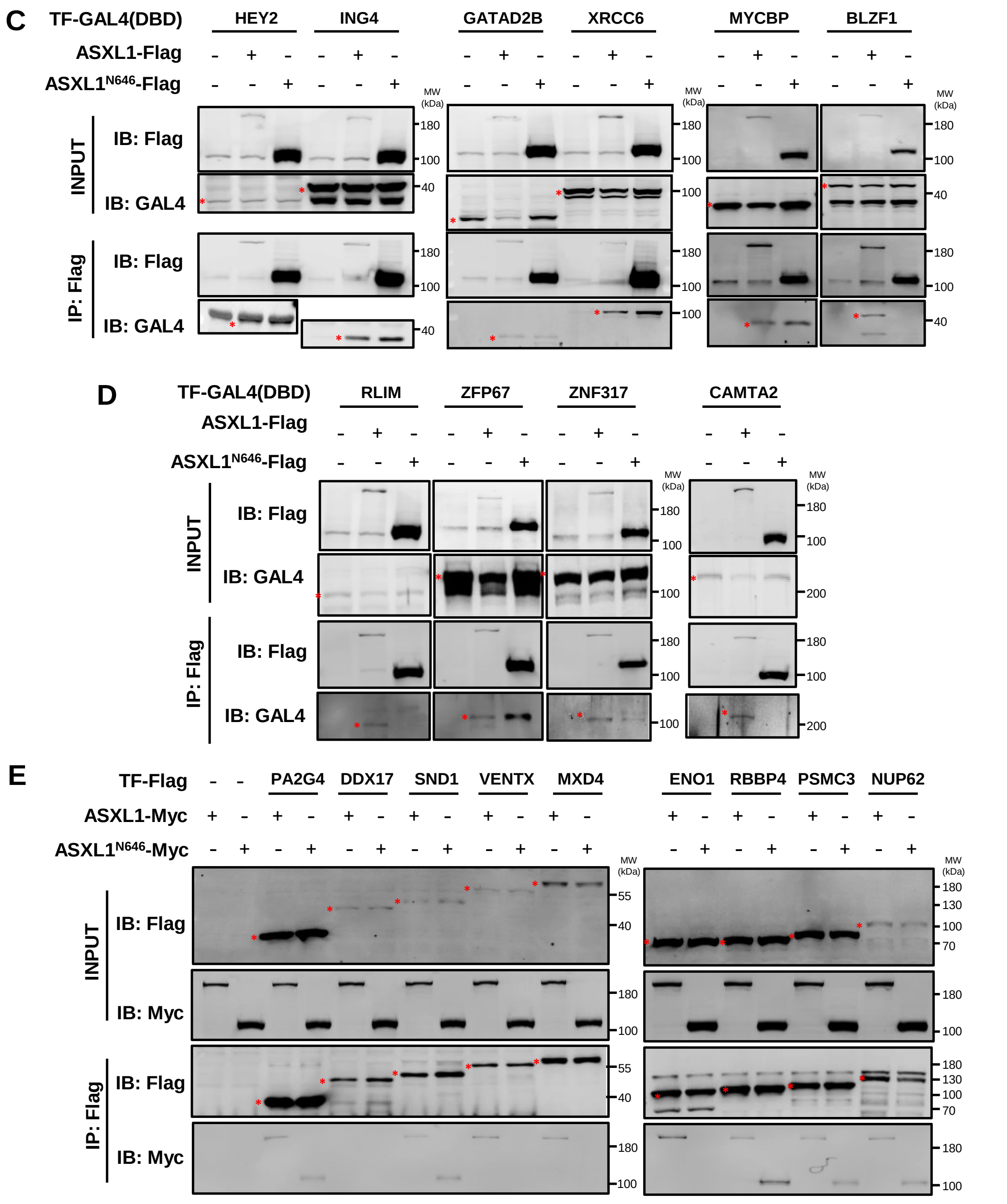

Figure S7. Identification of ASXL1 potential interacting transcription regulators

A, A summary of 17 DNA-binding proteins which were identified to be positive prey proteins for the mammalian two-hybrid system, and then were confirmed to interact with both full-length ASXL1 and $ASXL1^{N646}$ by western-blotting as shown below in (B-E).

B-E, Flag-tagged full-length or C-terminally truncated ASXL1 and GAL4(DBD)-tagged positive prey proteins were co-overexpressed in HEK293T cells, following IP with anti-Flag beads and western blotting to detect the protein interaction (B-D). Moreover, Flag-tagged positive prey proteins and Myc-tagged full-length or C-terminally truncated ASXL1 were co-overexpressed in HEK293T cells, following the same process to detect the protein interaction (E). The expected western blot bands are marked with red stars (*).

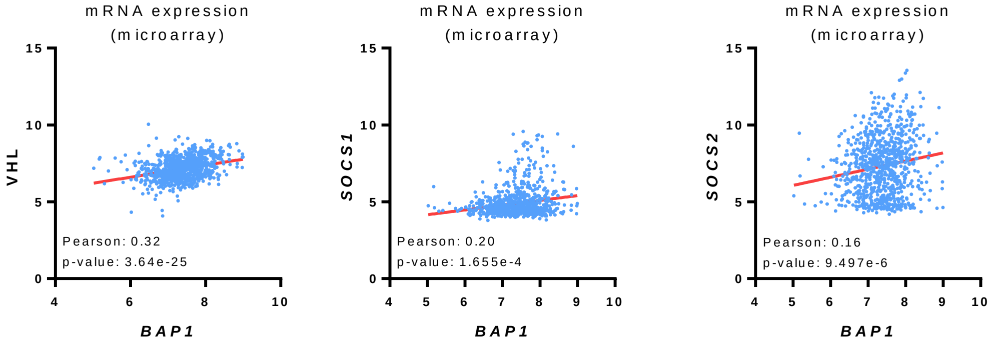

Figure S8. Positive correlation between the mRNA expression of *BAP1* and tumor suppressor genes *VHL* and *SOCS1/2* in different cancer cell lines

The expression of *BAP1* gene is positively correlated with the expression of *SOCS1* (p<0.001), *SOCS2* (p<0.001) and *VHL* (p<0.001). All the mRNA expression data was retrieved from Cancer Cell Line Encyclopedia dataset of cBioPortal database.

A

ChIP-Seq data source				
**Protein**	**Cell Line**	**Tissue**	**PMID**	**CistromeDB**
FOXK1	HEK293	Kidney	27705803	70731
FOXK2	K562	Bone Marrow	29126249	63744
ASXL1	HEK293	Kidney	27705803	76682
EZH2	DND-41	Blood	22955616	45192
SUZ12	K562	Bone Marrow	29126249	63519

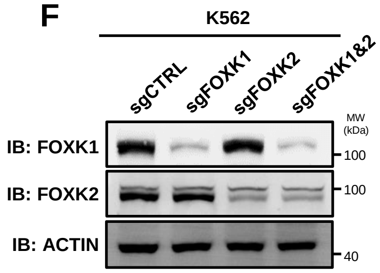

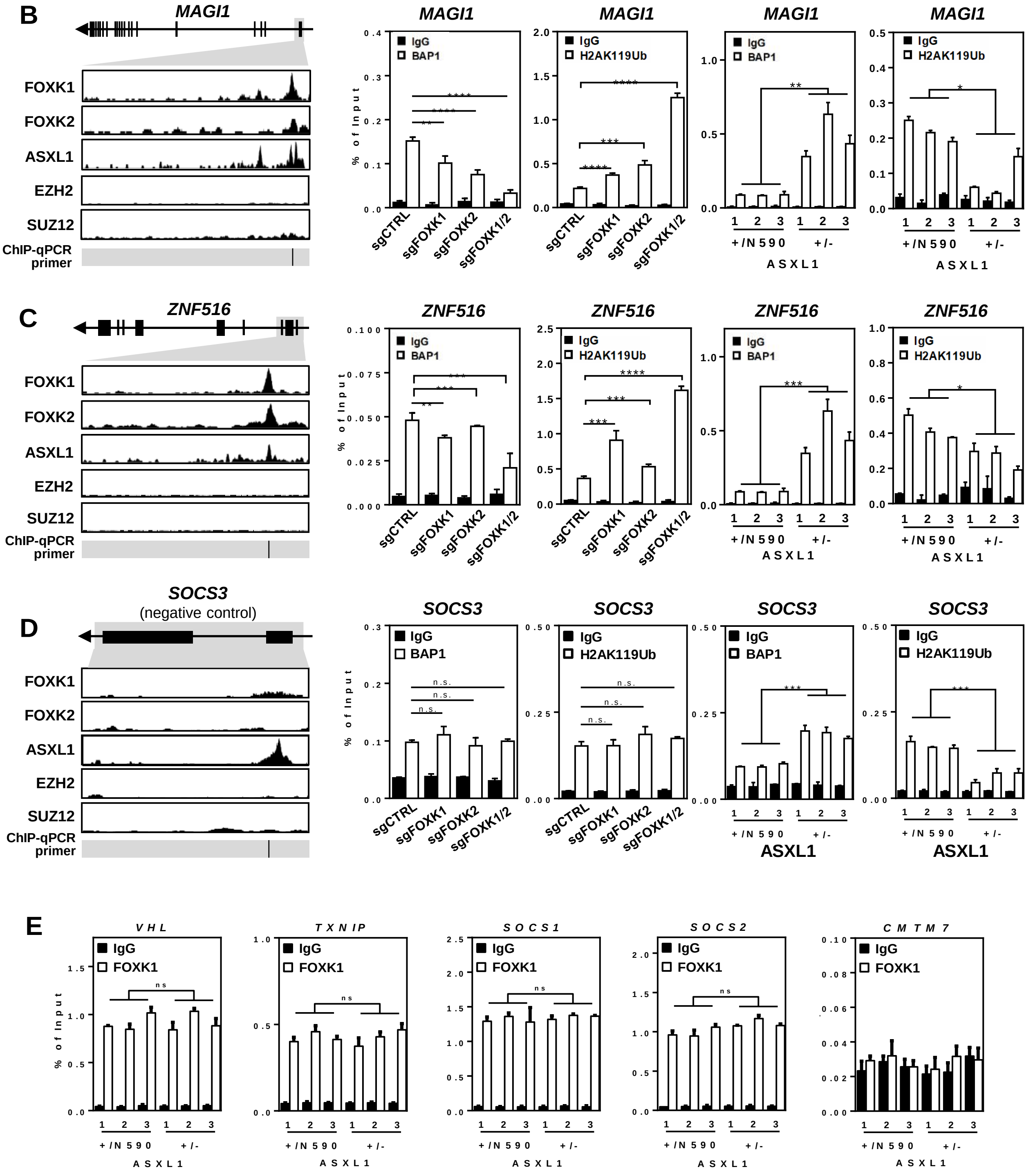

Figure S9. Tumor-derived ASXL1 mutant inhibits BAP1 binding to FOXK1/K2 target genes *MAGI1* and *ZNF516*

A, List of the ChIP-Seq data source (e.g. PMID and CistromeDB ID) used in this study.

B-D, The occupancy of FOXK1, FOXK2, ASXL1, EZH2 and SUZ12 at the promoter regions of indicated genes, analyzed by using the Cistrome Data Browser and UCSC genome browser. Moreover, BAP1 and H2AK119Ub enrichment at promoters of these genes were detected by ChIP-qPCR in ASXL1$^{+/N590}$ knockout K562 cells and FOXK1 and/or FOXK2 knockout K562 pools. IgG was included as negative control for ChIP-qPCR. According to ChIP-Seq retrieved from the GTRD database (http://gtrd.biouml.org/), *SOCS3* is not a direct target gene of FOXK1 and FOXK2, although BAP1 is enriched at the promoter region of *SOCS3*. Thus, *SOCS3* is included as another negative control, in addition to *CMTM7* as shown in Figure 4F.

E, FOXK1 enrichment at promoters of indicated genes in *ASXL1*$^{+/N590}$ and *ASXL1*$^{+/-}$ K562 clones, determined by ChIP-qPCR analysis.

F, Verification of *FOXK1* and/or *FOXK2* knockdown efficiency in *FOXK1* and/or *FOXK2* knockout K562 cell pools by western blotting using the indicated antibodies.

Data information: Shown are average values with standard deviation (S.D.) of triplicated experiments. Asterisks denote statistical significance with two-tailed Student‘s t-test or one-way ANOVA (B, C, D, E). * $P < 0.05$; ** $P < 0.01$; *** $P < 0.001$; **** $P < 0.0001$ for the indicated comparison; n.s.= not significant.

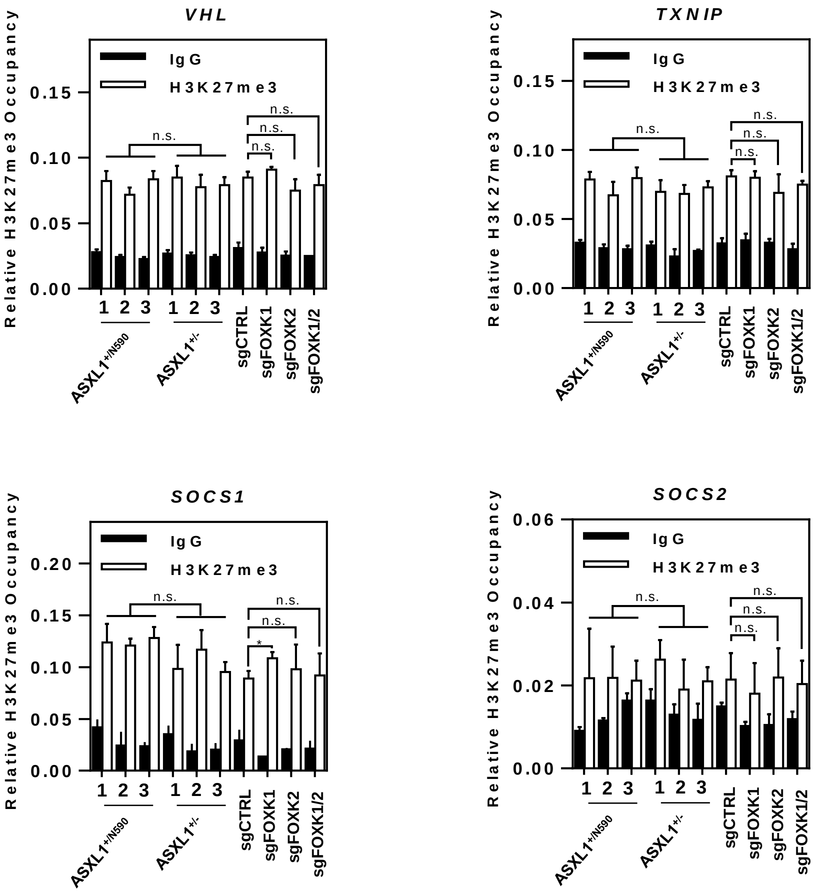

Figure S10. Tumor-derived ASXL1 mutant does not affect the H3K27me3 levels at the promoters of FOXK1/K2 target genes

Histone H3K27 trimethylation at the promoter regions of *TXNIP*, *VHL*, *SOCS1* and *SOCS2* genes in *ASXL1*$^{+/N590}$ and *ASXL1*$^{+/-}$ K562 clones, as well as FOXK1 and/or FOXK2 knockout K562 cells, as determined by ChIP-qPCR analysis.

Data information: Shown are average values with standard deviation (S.D.) of triplicated experiments. Asterisks denote statistical significance with two-tailed Student‘s t-test or one-way ANOVA. n.s.=not significant.

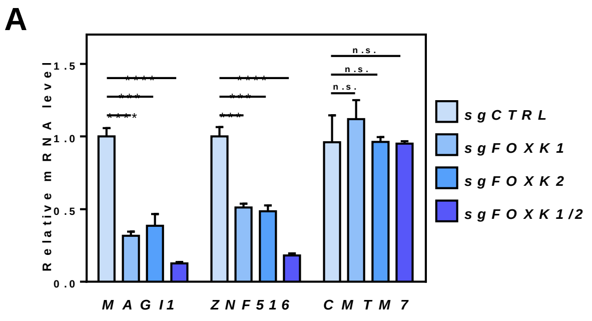

Figure S11. Tumor-derived ASXL1 mutant interferes with the BAP1-ASXL1-FOXK1/K2 transcription nexus to inhibit target genes *MAGI1* and *ZNF516*

A, The mRNA expression of *MAGI1, ZNF516*, and *CMTM7* in *FOXK1* and/or *FOXK2* knockout K562 cell pools, as determined by qRT-PCR.

B, The mRNA expression of *MAGI1, ZNF516* and *CMTM7* in *ASXL1*$^{+/N590}$ and *ASXL1*$^{+/-}$ K562 clones, as determined by qRT-PCR.

Data information: Shown are average values with standard deviation (S.D.) of triplicated experiments. Asterisks denote statistical significance with two-tailed Student's t-test (B) and with one-way ANOVA (A). ** P < 0.01; *** P < 0.001; **** P < 0.0001 for the indicated comparison; n.s.= not significant.

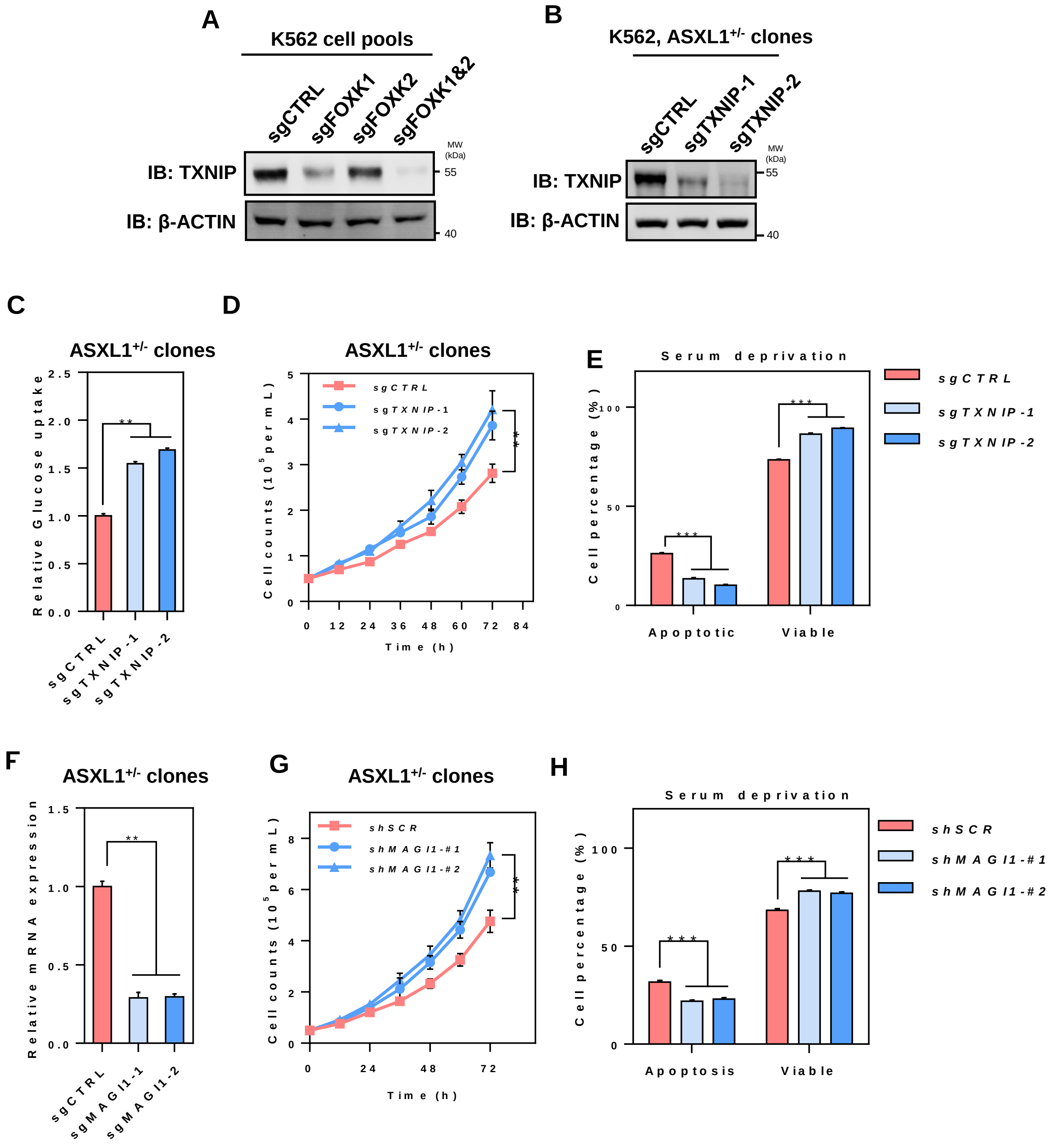

Figure S12. Depletion of *TXNIP* or *MAGI1* promotes leukemia cell proliferation and attenuates apoptosis

A, TXNIP protein levels in sg*FOXK1* and/or sg*FOXK2* K562 cells, as determined by western blotting using the indicated antibodies.

B, Verification of *TXNIP* knockdown in *ASXL1*$^{+/-}$ K562 cells by western blot analysis.

C, Measurement of glucose consumption in *ASXL1*$^{+/-}$ K562 cells with or without *TXNIP* knockdown by using the Glucose Assay Kit as described in the Materials and Methods.

Figure S12. Depletion of *TXNIP* or *MAGI1* promotes leukemia cell proliferation and attenuates apoptosis

D, Cell proliferation of *ASXL1*$^{+/-}$ K562 cells with or without *TXNIP* knockdown. After seeding into 6-well plates, 1x10^5 cells were incubated at 37° C for the indicated time periods. The cell numbers per well were determined at indicated time points as described in the Materials and Methods.

E, Cell apoptosis and viability of *ASXL1*$^{+/-}$ K562 cells with or without *TXNIP* knockdown under serum-deprived culture condition. Cells were maintained in RPMI-1640 medium without FBS for 48 hours, and cell apoptosis and viability were measured by flow cytometry as described in the Materials and Methods.

F, Verification of *MAGI1* knockdown efficiency in *ASXL1*$^{+/-}$ K562 cell clones by qRT-PCR.

G, Cell proliferation of *ASXL1*$^{+/-}$ K562 cells with or without *MAGI1* knockdown, determined as described above in (D).

H, Cell apoptosis and viability of *ASXL1*$^{+/-}$ K562 cells with or without *MAGI1* knockdown under serum-deprived culture condition, determined as described above in (E).

Data information: Shown are average values with standard deviation (S.D.) of triplicated experiments. Asterisks denote statistical significance with two-tailed Student's t-test (C, D, E, F, G, H). ** $P < 0.01$; *** $P < 0.001$ for the indicated comparison.

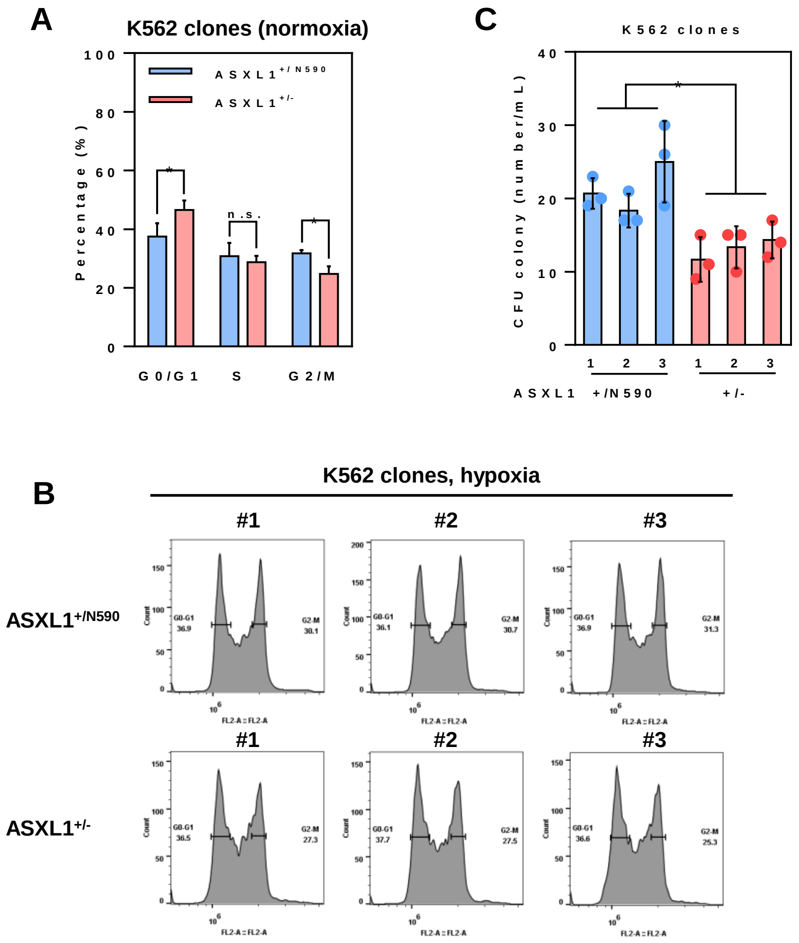

Figure S13. Tumor-derived ASXL1 mutant promotes the cell cycle exit from the G1 phase and enhances clonogenicity in leukemia cells

A, The ratios of G0/G1-phase and G2/M-phase in *ASXL1*$^{+/N590}$ and *ASXL1*$^{+/-}$ clones under normoxia condition for 24 hours. After staining with propidium iodide (PI), cells were analyzed by flow cytometer as described in the Materials and Methods.

B, *ASXL1*$^{+/N590}$ and *ASXL1*$^{+/-}$ K562 clones were cultured under hypoxia condition (1% O_2) for 24 hours. After staining with PI, cell cycle was analyzed by flow cytometer as described in the Materials and Methods. Histogram for the percentage of cells in each phase of cell cycle is shown in Figure 6C.

C, The capability of colony formation in *ASXL1*$^{+/N590}$ and *ASXL1*$^{+/-}$ clones. 300 cells were plated in a 6-well plate in the Methocult media supplemented with 10% FBS, and were incubated for 10 days for colony number counting as described in the Materials and Methods.

Data information: Shown are average values with standard deviation (S.D.) of triplicated experiments. Asterisks denote statistical significance with two-tailed Student's t-test (A, C), * $P < 0.05$ for the indicated comparison; n.s.= not significant.

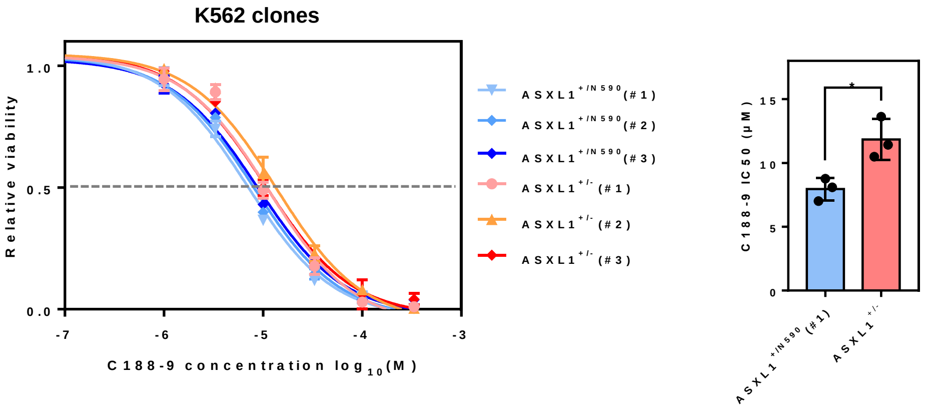

Figure S14. Tumor-derived ASXL1 mutant mildly increases the susceptibility of leukemia cells to STAT3 inhibitor

ASXL1$^{+/N590}$ and *ASXL1*$^{+/-}$ K562 clones were treated with the STAT3 inhibitor, C188-9, for 24 hours, and then the cytotoxicity was determined by CCK-8 assay as described in the Materials and Methods.

Data information: Shown are average values with standard deviation (S.D.) of triplicated experiments. Asterisks denote statistical significance with two-tailed Student‘s t-test, * $P < 0.05$ for the indicated comparison.
